# Beyond the bulk: disclosing the life of single microbial cells

**DOI:** 10.1093/femsre/fux044

**Published:** 2017-09-29

**Authors:** Katrin Rosenthal, Verena Oehling, Christian Dusny, Andreas Schmid

**Affiliations:** 1 Department Solar Materials, Helmholtz Centre for Environmental Research (UFZ), Leipzig, Germany; 2 Laboratory of Chemical Biotechnology, Department of Biochemical & Chemical Engineering, TU Dortmund University, Dortmund, Germany

**Keywords:** single cell analysis, heterogeneity, single cell dynamics, microfluidics, technical bias, lab-on-a-chip

## Abstract

Microbial single cell analysis has led to discoveries that are beyond what can be resolved with population-based studies. It provides a pristine view of the mechanisms that organize cellular physiology, unbiased by population heterogeneity or uncontrollable environmental impacts. A holistic description of cellular functions at the single cell level requires analytical concepts beyond the miniaturization of existing technologies, defined but uncontrolled by the biological system itself. This review provides an overview of the latest advances in single cell technologies and demonstrates their potential. Opportunities and limitations of single cell microbiology are discussed using selected application-related examples.

## Teaser

Analyzing single microbial cells reveals functionalities inaccessible via bulk-population studies. Studying individual cells requires special tools, but it also opens new horizons. Our current mechanistic understanding of individual cell behavior is still limited by available technologies.

## INTRODUCTION

What distinguishes a single and isolated microbial cell from a cell as a member of a microbial population or even a microbiome? The answer to this question is hidden in the cellular functionality of an isolated microbial cell. It is governed by cell internal parameters and the interaction of the cell with its immediate environment, without the influences of cell-to-cell interactions. Scaled down analytical technology and new lab-on-a-chip platforms are starting to reveal previously hidden phenomena in single cell physiology and metabolism. This provides new empirical and holistic access to microbiology and complements synthetic biology—which in turn is building up our understanding from the genome. Single cell studies provide new concepts in regulation and functioning of the genome, proteome and metabolome, inaccessible in bulk population studies. Concepts and discoveries from single cell studies can thus largely complement those from more classical approaches.

The single cell represents the basic functional unit in biology. The cumulative activity of cells comprises the measurable macroscopic output of microbial populations. What remains inaccessible with population analyses is the wide range of individual behavior, which can only be revealed by studying living single cells (Elowitz *et al.*^2002^; Lidstrom and Konopka 2010). Individuality is a fundamental feature of any cellular biological system and manifests in cell-to-cell heterogeneity (Lidstrom and Konopka 2010). Even isogenic microbial populations show extensive phenotypic heterogeneity among cells, evident from gene expression patterns, protein levels and metabolic activity (Mueller 2008; Heine *et al.*^2009^; Mueller, Harms and Bley 2010; Koch, Harms and Mueller 2014). The underlying mechanisms of cellular individuality are manifold (Ackermann 2015). Besides spontaneous genetic mutations, cell-to-cell differences arise from external perturbations or fluctuations in the cellular surroundings, which result in concerted physiological responses of the cell (Kussell and Leibler 2005; Acar, Mettetal and Van Oudenaarden 2008). Hence, cells will react heterogeneously when they experience individual differences in extracellular conditions due to, for example, spatial gradients (Wang *et al*. 2010b). However, a wide range of intracellular regulatory processes manifest in phenotypic heterogeneity despite homogeneous environmental conditions (Huh and Paulsson 2011). These processes comprise stochastic effects, multi-stability, regulatory oscillations, or partitioning of central control molecules with low abundance upon cell division (Jahn, Guenther and Mueller 2015). The biological importance of phenotypic heterogeneity has been attributed to increasing population fitness or survival chances upon environmental changes. The extent of phenotypic heterogeneity can also be relevant for efficient technical application of microbes (Arnoldini *et al.*^2012^; Arnoldini *et al.*^2014^; Delvigne and Goffin 2014).

**Figure 1.**
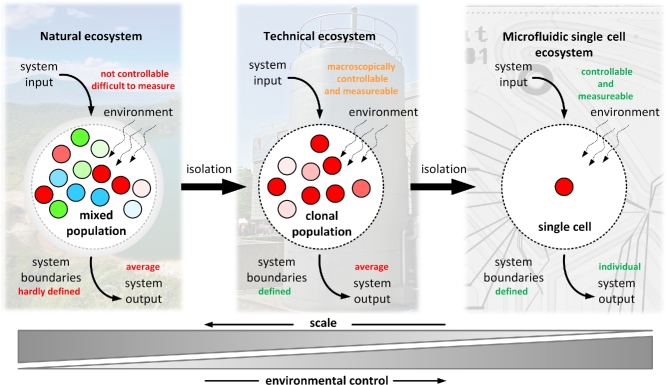
Single cell microbiology represents the final reductionist stage of microbiology and biotechnology. In natural ecosystems, system input and output cannot be quantitatively assigned to catalytic activity of individual community members or classes due to the open nature of the system. In population-based artificial ecosystems, system boundaries are defined and the catalytic activity can be linked to the genetic identity of the population, but again not to individual cells. In single cell ecosystems, environmental conditions can be stringently controlled and linked to single cell activity.

The identification and differentiation of biological mechanisms underlying phenotypic heterogeneity are very challenging. One example is the classification of phenotypic heterogeneity within populations in a quantitative manner. Due to the lack of a common mathematical formalism for describing heterogeneity, it proves difficult to compare results from different studies or experimental series (Delvigne *et al.*^2017^). This fact has to be addressed, for example by using simple biological key figures. The Gini coefficient, a parameter describing general heterogeneity, was recently proposed as a conceivable solution for describing the degree of phenotypic heterogeneity within microbial populations (Westerwalbesloh *et al.*^2017^). Our microbiological toolbox needs to be extended to address interdependent parameters and internal *vs* environmental control mechanisms of cellular functionality. This includes gradients, mass and energy transfer processes and their influence on physiology. New technologies are required to enable the quantitative analysis of single cells in controlled environments (Dusny and Schmid 2015a). Such emerging tools make use of tailored microfluidic environments, where individual cells can be cultivated with reduced bias due to chemical gradients or influences of neighboring cells. Microfluidic systems can be integrated in lab-on-a-chip platforms, enabling integral analysis of single cell physiology (Fritzsch *et al.*^2012^; Fig. 1). The application of technologies for analyzing single cells opens up fascinating opportunities. At the same time, as always, these technologies introduce bias due to the peculiarities of the created microhabitats. One has to exercise care to not overinterpret physiological data from single cells, as these might be artifacts from the artificial microhabitat. Carefully designed control experiments can remedy this and reduce the risk of false interpretations. Hence, we also outline current challenges, pitfalls and limitations of single cell technologies.

Individual cells can be studied in ‘single cell ecosystems’. Within the single cell ecosystem, the cell is uncoupled from the activity of surrounding cells by means of spatial isolation (Probst *et al.*2013b). The boundaries of the single cell ecosystem are technically defined by microstructures or microfluidic networks around individual microbes. The cells are put in place to facilitate the determination of the single cell system's input, output and dynamics (Rusconi, Garren and Stocker 2014). The exploitation of physics at the microscale allows control of the physicochemical properties of the extracellular environment to the level of bacterial cells (Weibel, Di Luzio and Whitesides 2007; Westerwalbesloh *et al.*^2015^). This control is of importance since it allows differentiating intrinsic from extrinsic factors, or determining origins of individual dynamics (Dusny *et al.*^2012^; Nikel *et al.*^2014^). Microfluidic concepts have mostly been developed to control individual cell microhabitats. An example is the prediction of mass transfer rates and substrate concentrations within microbial microcolonies with different microfluidic cultivation concepts via simulations (Westerwalbesloh *et al.*^2017^).

Microfabrication is now within reach of standard microbiological laboratories and requires only moderate financial and temporal investments. Mature and commercialized microfluidic technologies can be used and several template microbioreactor structures for soft lithography are available (Whitesides *et al.*^2001^; Qin, Xia and Whitesides 2010). (For further details on microbioreactor concepts for single cell microbiology, see the excellent reviews of Zare and Kim (2010) and Rusconi, Garren and Stocker (2014).) Despite useful microfluidic structures, the analytical and conceptual challenges for quantitatively analyzing cellular parameters of individual microbial cells are still immense (Schmid *et al.*^2010^; Fritzsch *et al.*^2012^; Dittrich and Jakubowski 2014). The handling of minute analyte volumes and amounts from single cells necessitates comprehensive adaptions of existing analytical tools. Recent analytics have become available that allow single cell genome, transcriptome or metabolome analysis (Kortmann, Blank and Schmid 2011; Fritzsch *et al.*^2012^; Vasdekis and Stephanopoulos 2015). Quantitative time-lapse microscopy, e.g. by applying fluorescent markers, is the most important and widely used analytical method for single cell analysis (Locke and Elowitz 2009). Optical approaches are simple to use and powerful, when the right markers or readouts are given. Physiological dynamics of single cells can be deduced from time-lapse microscopy imaging (Locke and Elowitz 2009). Cellular parameters can be measured that remain hidden in the bulk of a population, such as cell morphology. Many classical microbial physiological parameters like specific growth rates or production and uptake rates are not easily deduced from single cell studies (Gruenberger, Wiechert and Kohlheyer 2014; Dusny and Schmid 2015a,b). Yet, this is necessary for a holistic description of microbial parameters at a single cell level to complement population level studies.

The goal of this review is to provide the reader with an overview of the recent advances in single cell analyses, tools and concepts. We will focus first on studies linking cellular physiology with environmental physicochemical conditions, second on studies addressing biophysical properties of single cells, and third on those characterizing biochemical, metabolic and genomic aspects. Finally, we will describe specific environmental and technological aspects that are needed for single cell studies. Although we use many examples from bacterial single cell studies, the methods and concepts presented are not restricted to those. We do not cover technologies that analyze single cells in bulk populations. What emerges is a picture of single cells in isolation that gives us fascinating new insights into the properties and mechanisms of microbial physiology.

### Shaping the environment of single microbes

Single cell microfluidics can stringently control the cellular environment during cultivation and analysis with regard to nutrient and metabolite concentrations, osmolality, pH, shear force, temperature, or other physicochemical parameters (Ong *et al.*^2008^; Marques and Fernandes 2011).

For this the cells have to be kept in place during cultivation and analysis. A variety of methods for cell retention have been tested, comprising mechanical, hydrodynamic, electric, optical, acoustic, or magnetic cell manipulation (Fritzsch *et al.*^2012^; Lo and Yao 2015). Depending on the design of the microhabitat and the method of trapping, the experimenter can focus on one cell to obtain mechanistic insight or perform parallel experiments with hundreds of single cells. The underlying concepts have been described in detail elsewhere (Mustafi *et al.*^2012^; Benavente-Babace *et al.*^2014^; Mustafi *et al.*^2014^; Riordon *et al.*^2014^). Here we will focus on studies that retain single microbes in microstructured habitats for controlled perturbation experiments.

Perfusion enables the complete exchange of medium around isolated cells within seconds. This is a feature that cannot be realized with suspended microbial cultures and constitutes one of the most important aspects of microfluidic single cell analysis for analyzing cellular responses to environmental changes. Single cells can be trapped hydrodynamically in a microfluidic system and perfused with a fluid of defined chemical composition to trigger cell growth or division, perturbation, or metabolic production (Benavente-Babace *et al.*^2014^; Mustafi *et al.*^2014^; Riordon *et al.*^2014^). The principle of hydrodynamic trapping is based on the exploitation of altered fluidic resistances, which can be achieved by the creation of bypass channels with lower flow velocities or by sieve-like physical structures in microchannels (Benavente-Babace *et al.*^2014^; Riordon *et al.*^2014^; Khalili and Ahmad 2015). Many trapping structures restrict cell growth to a monolayer in one focal plane for optical analysis by matching channel heights and dimensions of microbial cells (Gruenberger *et al.*^2012^; Probst *et al.*2013a; Benavente-Babace *et al.*^2014^; Stratz *et al.*^2014^; Dusny *et al.*^2015^). Such microfluidic devices are particularly useful for massive parallelization of a large number of cell cultivations that can be observed optically. They have been used for measuring and tracking single cell responses to variations in medium composition in a time-resolved manner (Mustafi *et al.*^2012^; Gruenberger *et al.*^2013^; Probst *et al.*2013b; Unthan *et al.*^2014^). Differences in growth rate, gene expression or size change in single cells can be identified with high throughput. Single cell experiments with hydrodynamic trapping technologies provide statistically safe information on the heterogeneity of biological function among single cells and its interconnection to nutritional status or cell lineage. Further detailed examples will be described in the following sections.

The so called ‘mother machine’ is a prominent example of a hydrodynamic cell trapping structure. It consists of dead-end, cell-sized growth channels that are connected to a main feeding trench for medium supply and removal of surplus cells. Rod-shaped microbes are entrapped in the growth channels, allowing for division and cell elongation (Wang *et al.*2010a). Dynamics and fates of single mother and daughter cells can be followed before the daughter cells are displaced into the main trench. The mother machine has been used to study the robustness of single cell growth and cell size homeostasis (Wang *et al.*2010a; Jun and Taheri-Araghi 2015), physical and biochemical properties of chromosomes (Pelletier *et al.*^2012^; Youngren *et al.*^2014^), stochastic switching of motile cells into a chained, sessile state (Norman *et al.*^2013^) and dependency of cell wall growth on mechanical stress (Amir 2014) in *Escherichia coli* and *Bacillus subtilis.* Transient oscillations in constitutive gene expression and cell sizing could be analyzed in different *E. coli* strains during more than 20 000 individual cell cycles (Tanouchi *et al.*^2015^, ^2017^). In fact, the mother machine concept enables unique analyses of cell behavior, molecular partitioning, aging and cell fate in rod-shaped bacteria and fission yeasts. The continuous nature of the device, with supply of fresh medium and constant cell removal, allows long-term single cell growth experiments from days up to weeks for the first time. For example, growth regulation was shown to be robust in a single mother cell pole over hundreds of generations and decoupled from its replicative age.

One disadvantage of physical cell retention structures is that individual cells are influenced by surface contact or metabolic activity of neighboring cells, which can change their behavior (Geng *et al.*^2014^). Contactless cell trapping concepts have been developed to avoid this. One of the gentlest concepts for contactless trapping is cell manipulation by negative dielectrophoresis, which was originally developed for trapping larger mammalian cells, but was adapted for smaller microbial cells (yeast and bacteria). The weak electric field enables specific isolation and manipulation (alignment, isolation, trapping) of individual cells (Voldman 2006; Qian *et al.*^2014^). One particular embodiment (the so-called ‘Envirostat’ system, for environment–static) provides constant extracellular conditions for isolated cells via perfusion (Kortmann *et al.*2009a). The extracellular environment is shaped by laminar medium flow (Fritzsch *et al.*^2013^; Rosenthal *et al.*^2015^). The Envirostat concept enabled the determination of direct connections between the phenotypes of individual cells with their environmental conditions (Dusny *et al.*^2015^). It was found that isolated microbes respond to the constant microfluidic environment with higher specific growth rates than observed in populations (Dusny *et al.*^2012^). Using the Envirostat, a yeast-specific promoter system was proven to be ultrasensitive to carbon-catabolite repression. Repressing-sugar concentrations for the *MOX* promoter had hitherto been underestimated by almost four orders of magnitude within populations (Dusny and Schmid 2016). This accurate and quantitative description of promoter regulation could be achieved by decoupling cell and population activity with microfluidics.

Cells can also be manipulated contactlessly and isolated with optical tweezers using a focused laser beam (Zhang and Liu 2008). In contrast to negative dielectrophoresis, optical tweezers cannot be used for retaining and culturing single cells in isolation for longer time periods, because the high laser intensity induces heat and photodamage (Svoboda and Block 1994). Nevertheless, the combined application of optical tweezers and microfluidic cultivation is interesting, because a cell can be relocated to desired zones in the microfluidic system for further cultivation, analysis or enrichment (Wang *et al.*2011b; Probst *et al.*2013b). Umehara and coworkers followed growth of *E. coli* cells in microchambers and relocated daughter cells after cell division into spatially separated microchambers by using optical tweezers (Umehara *et al.*^2003^). Growth of the mother cell could be maintained for more than 90 h. It was observed that cells stopped elongation within 20 min independent of their cell cycle when changing from nutrient-rich to nutrient-free medium. The cells started to elongate again upon restoration of nutrient-rich medium within 30 min. This cycle was repeated three times and resulted in consistent adaptation dynamics of dividing cells, granting insight into the connection of growth and nutrient conditions.

The simplest method for single cell cultivation uses semi-solid growth supports such as agarose pads (Reinhard and van der Meer 2010). During cultivation on semi-solid agarose pads, cells are confined between the agarose surface and a glass coverslide. This entails a spatial restriction during cultivation, while nutrients diffuse from the agar to the cells (Young *et al.*^2012^). Agarose pads enable the simultaneous culturing of many cells, yet at the expense of limited cell isolation and lack of control of the cellular microenvironment (Dusny *et al.*^2015^). This cultivation concept was successfully applied for single cell-based toxicity assays and for cellular differentiation studies (e.g. Reinhard *et al*. 2013), and constitutes a convenient alternative in terms of throughput and accuracy to conventional but laborious and time-consuming 96-well-plate assays (Li *et al.*2014b). The spatial distances at which bacteria are typically spread on agarose pads can also be exploited for studying transfer processes between cells. This concept was used to investigate lateral gene transfer associated to integrating and conjugative elements in *Pseudomonas* (Reinhard *et al.*^2013^). Integrating and conjugative elements were found to induce host cell differentiation towards transfer competence in only a small proportion of cells. Limiting transfer competence to a few cells of the population was interpreted as being beneficial for the population fitness status, as integrating and conjugative element horizontal transmission is thus associated with little cost in terms of vertical transmission. This was a previously unknown mechanism for controlling host properties via transferable DNA elements to facilitate horizontal gene transmission.

Dynamics in inhibition of single cells were studied with agarose pads capped with polydimethylsiloxane covers with imprinted microfluidic channels (Li *et al.*2014a,b). The channels contained different solutions that diffused through the agarose pad and created a concentration profile. *Escherichia coli* cells were positioned on the agarose surface between source and sink channels and were monitored via microscopy to measure time- and concentration-dependent inhibitory effects of antibiotics on growth (Li *et al.*2014b). Additional data such as concentration-dependent morphological changes such as filamentation and bulge formation were collected. Furthermore an opportunistic persistence was observed, meaning that inhibited *E. coli* cells benefited from lysed cells in close proximity, recovered and started to re-grow (Li *et al.*2014a,b). Given the simplicity of basal cultivation systems such as agarose pads, labor can be focused on revealing biological mechanisms instead of on the sometimes tedious design and implementation of complex microfluidics. However, this only applies to cases where the envisaged studies do not require the stringent control of the cellular surrounding.

Every microsystem has its specific and distinct degrees of analytical freedom. Agarose pads and some of the mentioned hydrodynamic systems allow parallel investigation of many individual microbial cells. This is achieved at the expense of tight environmental control. Single cell perfusion methods, such as the Envirostat technology, have limited throughput, but can be used to more specifically manipulate and control individual cells, enabling mechanistic studies uncoupled from the activity of other cells (Kortmann *et al.*2009b; Rosenthal *et al.*^2015^). However, single cell ecosystems are artificial, and possible biases caused by the specific retention method or by interference of the materials used for constructing the microfluidic device have to be considered carefully.

### Environmental impacts on cellular physiology

**Figure 2.**
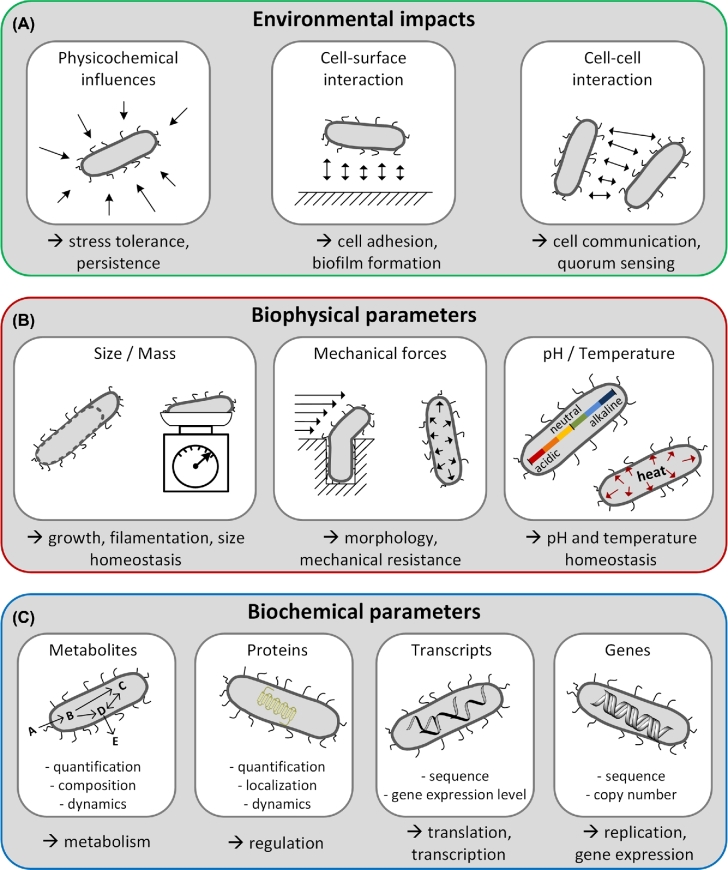
Current broad aspects of single cell research. (**A**) Studies focusing on environmental impacts, such as adhesion or signaling. (**B**) Studies focusing on biophysical parameters of single cells, such as size, mass and mechanical properties. (**C**) Studies of biochemical aspects, such as single cell metabolites, proteins or genetics.

Single cell cultivation technologies enable linking cellular physiology with controlled extracellular physicochemical conditions. Extracellular factors influencing the cell's behavior include, for instance, cell-surface interactions and cell-to-cell interactions. Technologies and corresponding application examples are reviewed next (Fig. 2A).

#### Cell-to-cell interactions

In both natural and artificial ecosystems, microbial cells are in close contact and continuously interact with each other (Fig. 2A; Li and Tian 2012; Neu and Lawrence 2015; Schlafer *et al.*^2015^). Cell-to-cell interactions comprise sharing metabolites, excreting defense molecules and synchronizing physiological activities (Caro *et al.*^2007^; Stepanauskas 2012). Such cell-to-cell interactions were studied with agarose pads with imprinted parallel sub-micrometer growth tracks (Moffitt, Lee and Cluzel 2012). Inserted cells grew in the tracks and outgrowing cells were flushed into a gutter trench. As an example of a symbiotic relationship, growth of two *E. coli* mutants, each auxotrophic for different amino acids, was followed in parallel tracks. Secreted amino acids diffused through the porous agarose sidewalls of the channels, which allowed mutual exchange of essential metabolites (Moffitt, Lee and Cluzel 2012). The elongation rate of single *E. coli* cells was dependent on the culture composition and on the spatial distances between both auxotrophic mutants. Auxotrophs separated by distances of less than ∼20 μm grew 3- to 5-fold faster than cells separated by longer distances (Moffitt, Lee and Cluzel 2012). This example has implications for cell-to-cell metabolic interactions and mass transfer for establishing symbiotic lifestyles.

Cell–cell communication by quorum-sensing (QS) and its physiological consequences can be excellently studied at the single cell level (Waters and Bassler 2005; Keller and Surette 2006). QS enables a collective, multicellular organism-like behavior of the population (Bassler and Losick 2006). It is regulated by extracellular signaling molecules called autoinducers. Their levels correlate with cell densities in populations and cells alter gene expression when the autoinducer concentration exceeds or falls below a certain threshold (Waters and Bassler 2005). Examples of some QS-regulated processes are the production of virulence factors or antibiotics, exoproteolytic activity, biofilm formation, bioluminescence production and swarming motility (Hammer and Bassler 2003; Waters and Bassler 2005; Anetzberger, Pirch and Jung 2009; Long *et al.*^2009^; Perez and Hagen 2010; Anetzberger, Schell and Jung 2012; Castillo-Juarez *et al.*^2015^).

Single cell technologies are useful for understanding the mechanistic principles of QS. *Pseudomonas aeruginosa* cells have been captured in aqueous droplets for analysis of the variability of QS (Boedicker, Vincent and Ismagilov 2009). The droplets were generated by pumping a suspension with low cell density through a microfluidic channel with tiny wells. Subsequently, an air bubble was introduced that removed excess liquid and formed individual aqueous droplets with a volume of merely 100 fL per well. Each droplet contained one cell or a small number of cells (max. 14) and QS sensing was monitored by a genetically encoded fluorescence reporter (Hentzer *et al.*^2002^). The initiation of QS was found to be highly variable among *P. aeruginosa* cells and even single cells were able to initiate QS on their own when the droplet volume was small enough (Boedicker, Vincent and Ismagilov 2009). QS communication between two cells was monitored with cells trapped in double droplets (Bai *et al.*^2013^). A monodisperse emulsion of droplets was created with a microfluidic droplet generator (Bai *et al.*^2010^). Droplet pairs were confined in traps to form a droplet interface bilayer, which enabled the diffusion of molecules between the droplets (Bai *et al.*^2013^). Two recombinant *E. coli* strains were investigated, which either secreted or sensed the autoinducer *N*-(3-oxododecanoyl)-L-homoserine lactone (OdDHL) (Andersen *et al.*^2001^; Bai *et al.*^2013^). OdDHL sensing was detected with a genetically encoded fluorescence reporter (Andersen *et al.*^2001^). The pair of droplets with an OdDHL-producing cell in one and an OdDHL-sensing cell in the other was trapped and successful induction of QS was detected upon diffusion of OdDHL across the droplets. With this approach, intra-species QS was proven at the single cell level for the first time. Unfortunately, these droplet technologies for studying cell–cell interactions are currently not applicable for high throughput as they are limited to the simultaneous analysis of maximally two droplets.

**Figure 3.**
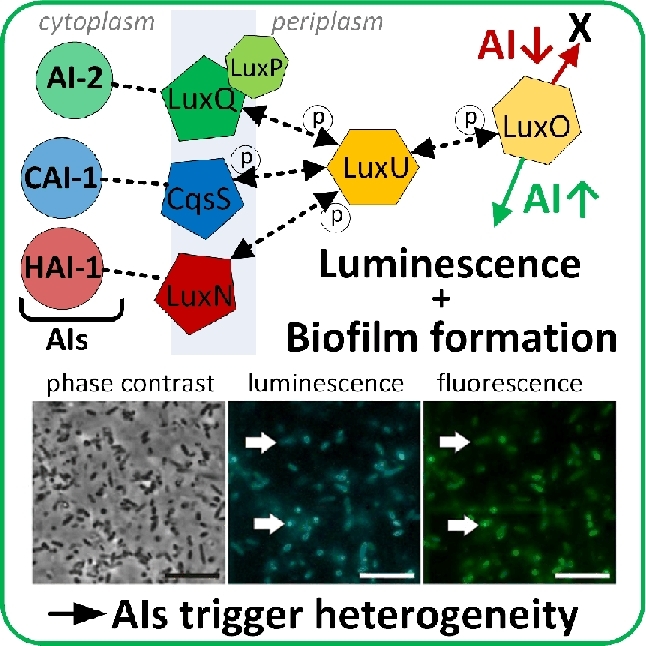
Single cell heterogeneity as a response to auto inducer molecules (AIs). The images show bioluminescent cells of *V. harveyi* that respond to the presence of AIs with bioluminescence and biofilm formation. The length of the scale bar is 2.5 μm. (Adapted from Anetzberger, Schell and Jung 2012 with permission from BioMed Central.)

Microscopy studies of individual cells in growing populations of bioluminescent *Vibrio harveyi* and *Vibrio fischeri* revealed QS heterogeneity (Anetzberger, Pirch and Jung 2009; Perez and Hagen 2010; Plener *et al.*^2015^). QS in *V. harveyi* and *V. fischeri* is regulated by the *lux* operon (Fig. 3; Anetzberger, Schell and Jung 2012) leading to bioluminescence as a direct output of the *lux* regulatory cascade (Plener *et al.*^2015^). Light intensities varied between individual cells and variability increased with increasing cell densities. Individual cells of cultures with a high cell density exhibited increased bioluminescence compared with those from cultures with low cell densities. Furthermore, bioluminescence was more heterogeneous in the case of *V. harveyi*, which also produced more biofilm (Anetzberger, Pirch and Jung 2009). Thus, QS is related to individual bioluminescence and biofilm formation. Autoinducers were also found to be involved in the formation of heterogeneous gene expression in clonal populations of *V. harveyi* (Fig. 3; Anetzberger, Schell and Jung 2012). Expression of *luxC*, the first gene of the fatty acid reductase complex of the *lux* operon, was quantified by fluorescence microscopy targeting several bioluminescence-related genes fused to the green fluorescent protein gene. The number of cells expressing *luxC* increased over the cultivation period. Furthermore, induction of *luxC* expression of individual population members was heterogeneous (Anetzberger, Schell and Jung 2012). The knowledge obtained about QS mechanisms can be utilized to manipulate microbial communication. This is particularly important to avoid one species taking over control in a microbial multispecies community, e.g. in a number of bacterial pathogenic processes (Vikram *et al.*^2011^) or controlling population compositions in waste water treatment (Lade, Paul and Kweon*.*2014a,b).

#### Tolerance and adaptation

Mechanisms for adaptation during environmental stress exposure ensure the survival of microbial populations. Bet-hedging is a mechanism that involves stochastic switching of phenotypes, and the transition between phenotypes is actively induced upon recognition of a stress signal (Beaumont *et al.*^2009^). A bimodal on/off-switching of gene expression supports the formation of two subpopulations, and the switching rate is variable among individual population members (Balaban *et al.*^2004^; Acar, Mettetal and Van Oudenaarden 2008; Nikel *et al.*^2014^). The resistant subpopulation has a higher survival probability when environmental perturbations occur. A more complex counter-mechanism to stress was reported for eukaryotic cells based on switching among several phenotypes (Levy, Ziv and Siegal 2012). Various subpopulations are formed prior to the appearance of a stress signal due to stochastic gene expression and deterministic factors, which results in at least a small fraction of stressed cells that are able to survive (Balaban *et al.*^2004^; Nikel *et al.*^2014^; Martins and Locke 2015).

As an example for bacterial microorganisms, *Streptococcus pneumoniae* has evolved several co-existing phenotypes that enable it to resist the effect of antibiotics (Sorg and Veening 2015). Exposure of *S. pneumoniae* cultures to eight different bacteriostatic or bactericidal antibiotics resulted in distinct inhibition profiles: the cells grew unimpaired for certain time periods after exposure to the bactericides, but the periods of unimpaired growth became shorter as the bactericide concentrations increased. Some of the cells responded with a complete shutdown of gene expression activity, and a large cell fraction was irreparably damaged or died during growth arrest. The addition of bacteriostatic compounds led to reduced growth velocities. Growth-arrested cells exhibited higher metabolic activities after exposure to bacteriostatic antibiotics than untreated cells. Bacteriostatics and bactericides provoked different types of adaptation mechanisms, indistinguishable at a population scale. The recovery times of cells exposed to bacteriostatics (indicated by longer lag phases) scaled with exposure time. The extended lag phases of the cultures treated with bacteriostatics were associated with increased phenotypic heterogeneity. Cells whose parental cell metabolism had adapted to the bactericide cephalexin displayed a lower susceptibility to this antibiotic. These cells survived and resumed growth although most cells died after cephalexin treatment (Sorg and Veening 2015). It thus appears that pre-adaptation of the culture to a certain antibiotic might facilitate stochastic switching between phenotypes. We learn from these examples that cellular responses to stress cannot be fully resolved with suspended cultures.

**Figure 4.**
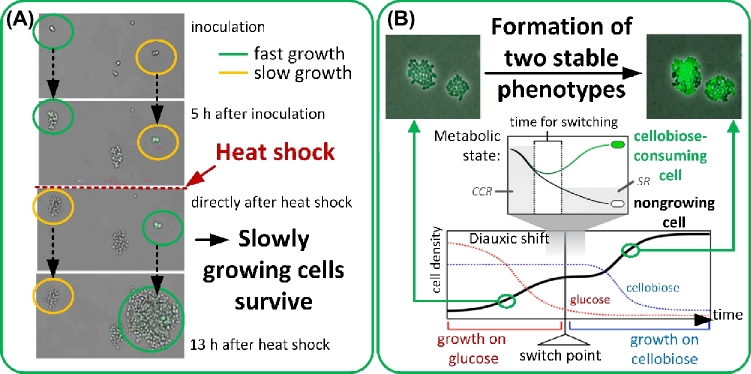
Growth arrest in single cells as a survival strategy under stress. (**A**) Cells of *S. cerevisiae* with different growth phenotypes. Slow growing cells survive a heat shock and take over the population after termination of the heat treatment (70 min at 60°C) (adapted from Levy, Ziv and Siegal 2012). (**B**) Heterogeneity in growth of individual *L. lactis* cells as a bet-hedging mechanism. Upon change of the carbon source, the rearrangement of the metabolism to the new substrate is dependent on the available energy (metabolic state). Cells with high levels of available energy overcome the regulatory burden of carbon catabolite repression (CCR) much faster than cells with a low available energy. (Adapted from Solopova *et al*. 2014.)

Phenotypic heterogeneity in *Saccharomyces cerevisiae* was beneficial for the survival of heat stress (Levy, Ziv and Siegal 2012). Gene expression levels of the regulator protein Tsl1were variable population-wide and responsible for counteracting high temperatures. Tsl1p is a trehalose-synthesis regulator that is part of the general stress response of *S. cerevisiae* (Winderickx *et al.*^1996^; Singer and Lindquist 1998). Slowly growing cells produced higher amounts of the regulator protein and survived heat shock (Fig. 4A). The population-wide variance in the regulator protein level during stress thus confirmed the benefits of phenotypic intrapopulation heterogeneities for survival during stress (Levy, Ziv and Siegal 2012).

The investigation of individual cellular responses to environmental stress has enabled redefinition of the origin of lag phases during diauxic shifts from one substrate to another (Boulineau *et al.*^2013^; Solopova *et al.*^2014^; Stratford *et al.*^2014^). Classical population-based theory of diauxic growth teaches that all cells in the population rearrange their metabolism to adapt their enzymatic machinery to a new carbon source upon depletion of the preferred carbon source (Monod 1949). Populations continue to grow exponentially after metabolic rearrangement (Monod 1949). Bet-hedging mechanisms were identified as a further possible reason for prolonged lag phases of populations during diauxic growth. Kotte *et al.* (2014) reported the formation of two subpopulations of an isogenic *E. coli* population with either a growing or a non-growing/dormant phenotype after switching from glucose to gluconeogenetic carbon sources. In addition, experiments with *Zygosaccharomyces bailii* (Stratford *et al.*^2014^), as well as *Lactococus lactis* (Fig. 4B) (Solopova *et al.*^2014^) and *E. coli* (Boulineau *et al.*^2013^) demonstrated that growing cells were pre-equipped to metabolize the new carbon source. These subpopulations continued to grow with specific growth rates similar to those of the population prior to the shift of growth substrates, while the bulk of the population members stopped growth or died owing to the shift. The lag phase observed at a population scale was therefore an artifact due to the delay in monitoring biomass and not a biological response itself.

A recent example demonstrated the protective role of loosely controlled, so-called noisy response of cells to stress and its role for survival during a subsequent stress (Mitosch, Rieckh and Bollenbach 2017). It was found that antibiotic treatment triggered the noisy expression of the *gadBC* acid resistance operon in single *E. coli* cells. With microfluidic shift experiments, a clear link between enhanced survival during acid stress, *gadBC* expression and a previous antibiotic treatment could be identified. The cross-protection between antibiotics and other stressors could hence be uncovered.

Single cell cultivation systems can also be used as powerful screening tools for identifying mutants with beneficial phenotypes at high throughput and accuracy. Individual mutants of the cyanobacterium *Synechococcus elongatus* PCC 7942 with beneficial phenotypes, especially a high tolerance against alcohols, were recently identified by Arai *et al.* (2017) using a single cell-based screening concept. *Synechococcus elongatus* PCC 7942 is known to have a generally low tolerance towards alcohols and has consequently not been applied in the production of photobioalcohol from sunlight and CO_2_. Via UV-C-induced random mutagenesis, a mutant library was established and enriched in medium containing 10 g L^−1^ isopropanol. A subsequent single cell-based microarray cultivation step enabled identification of the fastest growing mutants (Arai *et al.*^2016^). The strain finally isolated was able to grow in the presence of up to 30 g L^−1^ isopropanol and showed increased tolerance towards other alcohols such as ethanol, 1-butanol, isobutanol and 1-pentanol. This strain is currently under investigation as a potential next-generation biocatalyst for the high level production of alcohols from CO_2_.

#### Cell surface interactions

Microorganisms interact with surfaces in their surroundings. In nature, an immense number of microorganisms thrive on solid surfaces or interfaces as biofilms (Vigeant *et al.*^2002^). Biofilm formation allows the physiological interaction of several species in spatially close proximity, which can be either competitive or cooperative. (For more information about biofilm formation and social interactions within biofilms see the excellent reviews of Wimpenny, Manz and Szewzyk (2000) and Li and Tian (2012).) Biofilms protect cells from extracellular influences, which can be critical in biofilm-related bacterial infections (Wu *et al.*2015b). Biotechnological production processes with solvents benefit from biofilms due to their increased resistance compared with suspended cells (Halan, Buehler and Schmid 2012).

The initial cell attachment mechanism and the formation of a stable biofilm are central research topics in single cell microbiology. To evaluate the involvement of electrostatic, van der Waals and hydrodynamic forces in the cell attachment mechanism, distances between cells and solid surfaces before cell attachment have been quantified (Berke *et al.*^2008^; Lauga *et al.*^2006^; Vigeant *et al.*^2002^). A cell suspension was introduced between two glass coverslips and the attraction of motile cells to surfaces was determined via total internal reflection fluorescence microscopy at high optical resolution (Lauga *et al.*^2006^; Berke *et al.*^2008^). The probability of cell adhesion and biofilm formation increased when cells stayed near surfaces (Vigeant *et al.*^2002^). Electrostatic and van der Waals forces were found to be responsible for the initial, irreversible cell attachment, as these forces are known to have an effect in short ranges of ∼50 nm. In contrast, hydrodynamic forces range over several micrometers and were identified as keeping the cells in proximity to the surfaces (Vigeant *et al.*^2002^). With this knowledge, microbial colonization of solid surfaces can now be understood from its initial stages on. Studying physical interactions of isolated living microbes with matter might lead to new perspectives on the initiation of surface-attached lifestyles such as microbial biofilms in natural and technical environments.

Lin, Crosson and Scherer (2010) analyzed the swarming motility and surface adhesion mechanisms of single *Caulobacter crescentus* cells within a microfluidic device by microscopy. The device consisted of a rectangular polydimethylsiloxane flow channel attached to a glass coverslip. Adhesion frequencies were found to be dependent on expression levels of the multiple actuator *divJ*. The actuator codes for a histidine kinase (EC 2.7.13.3) that regulates cell division and differentiation (Wheeler and Shapiro 1999). High DivJ levels inhibited surface adhesion and decreased swarming in semi-solid media. Swimming motility, analyzed by measuring the cell swimming speed in micrometers per second, was not impeded by high DivJ concentrations and was thus excluded as a cause for the observed swarming limitation (Lin, Crosson and Scherer 2010).

The assembly and composition of cell surfaces and their biophysical properties have been investigated in order to disclose cell adhesion mechanisms (Dufrêne 2014). Cell surface properties were studied using atomic force microscopy, in which samples are scanned with a nanoscale tip attached to a flexible cantilever. The movement of the cantilever was mapped with a reflected laser beam and translated into a three-dimensional topological image with nanometer resolution (Dufrêne 2002). Several surface structures of cells were described at a high resolution using atomic force microscopy. By localizing cell wall components, conclusions on their functionality and physiological role could be drawn (Andre *et al.*^2010^, ^2011^). It was found that the heterogeneous distribution of teichoic acids in the outer cell wall of *Lactococcus plantarum* controls cell division (Dufrêne 2014). Furthermore the number of flagella of *Bacillus thuringiensis* directly correlated with their swarming motility (Gillis *et al.*^2012^). Atomic force microscopy was further developed to single cell force spectroscopy by replacing the atomic force microscopy cantilever tip by a cell (Benoit and Gaub 2002). The immobilization of microbes to the atomic force microscopy cantilever can be applied with, for example, adhesive (Zeng, Mueller and Meyer 2014). This is a quick and easy method; the simultaneous immobilization of several cells cannot, however, be excluded and the precise positioning of cells on the cantilever is challenging. Beaussart *et al.* (2013) improved the attachment of cells by bonding polydopamine-coated colloids to the cantilever and immobilizing single cells to the colloids. This prevented multiple cell attachment, cell surface denaturation and cell loss due to weak cell-cantilever bonding. Furthermore, the use of coated colloids for cell bonding reduced cell damage due to heat transfer caused by the laser beam. Individual *Lactobacillus plantarum* cells were immobilized with this technique to measure the adhesion forces between cells and surfaces. Interestingly, the strength of bonding of *L. plantarum* to a hydrophobic (abiotic) surface was time-independent, while bonding to lectin (biotic) surfaces got stronger over time. This observation could be attributed to glucose-based polysaccharides on the cell surface that caused slower formation of lectin bonds (Beaussart *et al.*^2013^). Hence, adhesive properties of individual cells on defined surfaces can be easily quantified in order to identify adhesion influencing parameters.

### Single cell biophysics

Technologies for the quantification of biophysical cellular parameters are required for describing global physiological functions and mechanisms such as growth, morphology, regulation and homeostasis (Fig. 2B). In the following section, we review recent technologies for measuring biophysical cellular parameters such as size, mass, morphology, mechanical forces, pH and temperature of individual microbes.

#### Cell growth

Microbial growth directly depends on to the conditions prevailing in the cells’ microenvironmental surroundings, as well as the intracellular constitution (Schaechter 2015). It unrestrictedly reflects physiological changes, e.g. in gene expression patterns, medium compositions, cell sizes and ribosome concentrations per cell, with minimal delay (Scott *et al.*^2010^; Klumpp and Hwa 2014; Schaechter 2015). Growth is thus particularly interesting as a global physiological readout. Microbial growth on the population scale is typically analyzed by measuring the increase of the optical density of a cell suspension over time or by automatic high-throughput cell counting in a coulter counter (Bryan *et al.*^2012^). This enables determination of specific growth rates of whole populations, whereby dynamics in individual growth rates are pooled. Individual cell growth can be analyzed and quantified by applying distinct single cell analysis methods. These methods are based on the determination of the cell number, cell size (volume, area, elongation) and cell mass.

Growth of small micropopulations can be described by cell numbers determined by cell counting. Computer-assisted processing of microscopy images even enables automatic quantification of cell numbers (Probst *et al.*2013b). Growth from initially one bacterium up to microcolonies consisting of more than 500 cells was followed by applying this method. Cell counting can also be used for analyses on a population level. Cell counting is thus a suitable method for comparing population- and single cell-based results (Gruenberger *et al.*^2013^; Unthan *et al.*^2014^). Unthan *et al.* used cell counting at single cell and population levels and revealed an iron-chelating medium compound as a factor for biphasic growth during bioreactor cultivation of *Corynebacterium glutamicum* in defined medium.

However, the analysis of cell growth via cell counting is based on the assumption that all considered cells are similar in size and length. This is probably only true for cells in constant cultivation conditions (Probst *et al.*2013b). Additionally, cell counting is limited to growth analyses of microcolonies or populations, because growth of individual cells does not affect cell numbers.

**Figure 5.**
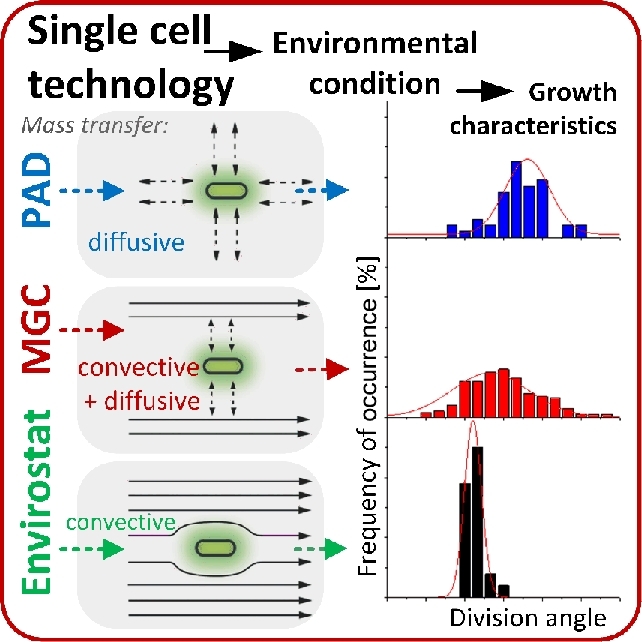
Technological concepts for producing controlled single cell environmental conditions. The examples illustrate three distinct technologies for single cell microbiology and their respective properties in terms of mass transfer: semi-solid agarose pad (PAD), microfluidic monolayer growth chamber (MGC) and non-contact traps driven by negative electrophoresis (Envirostat). Growth studies with *C. glutamicum* revealed that the different microenvironmental conditions with these technologies determine growth characteristics such as division angles and division times. (Reproduced from Dusny *et al*. 2015 with permission from the Royal Society of Chemistry.)

Quantitative measurements of cell geometry, such as cell volume, enable the following of growth of a cell over time, even if it is not dividing. The growth behavior of individual cells can thus be quantitatively compared. A straightforward approach to measure total cell volume is the manual determination of cell geometry from microscopy images. This method was shown to be universally applicable for the calculation of single cell growth rates of microbes that were cultivated in different microfluidic structures (Dusny *et al.*^2012^, ^2015^). Semi-solid agarose pads, microfluidic monolayer growth chambers and non-contact cell traps driven by negative electrophoresis (Envirostat) were applied for single cell cultivation (Fig. 5), which provided distinct environmental conditions. Individual *C. glutamicum* cells were isolated from an exponentially growing population. The investigated cells exhibited similar specific volumetric growth rates independent of the cultivation technology used. Specific volumetric growth rates of isolated cells were equal to or even higher than in population cultivations (Dusny *et al.*^2015^). This demonstrates that maximal specific growth rates obtained in population-based ecosystems do not reflect the maximal possible growth rates of cells that experience optimal growth conditions. Morphological aspects such as division rates, division angles and division symmetry of cells were inconsistent in the three devices. The morphology of cells grown on semi-solid agarose pads significantly differed from cell morphologies cultivated in the monolayer growth chambers and the Envirostat (Dusny *et al.*^2015^). The authors attributed those differences to spatial constriction, local substrate/nutrient depletion and accumulation of inhibiting products in semi-solid agarose pads. Hence, non-optimal growth conditions were better compensated by the regulation of the specific growth rate than of the cellular morphology (Dusny *et al.*^2015^). This study demonstrates that growth and morphology are directly dependent on the environmental conditions around the individual cell. This suggests a deliberated choice of cultivation technology, as every technique imposes technology-specific conditions on the cell investigated.

The laborious and time-consuming manual determination of cell volumes limits the applicability of this method in terms of time. Automated segmentation is faster, but requires computational solutions for image processing, which are prone to error. Most of the available analysis software solutions, such as Schnitzcells or MicrobeTracker, are limited to certain cell types as they are based on cell segmentation with defined morphological boundary conditions (Sliusarenko *et al.*^2011^; Young *et al.*^2012^; Chowdhury *et al.*^2013^). The detection of cell areas at high precision is especially reliable for rod-shaped bacteria, because most software algorithms are adjusted to the specific morphology of common laboratory strains (Sliusarenko *et al.*^2011^; Young *et al.*^2012^). Many industrially relevant bacteria are rod-shaped, such as *E. coli*, *C. glutamicum*, *B. subtilis* and *Pseudomonas* sp., but many other morphological manifestations of microorganisms exist that are not quantifiable with common software packages. In this sense, the recently developed image analysis software Oufti is particularly worth mentioning (Paintdakhi *et al.*^2016^). Oufti allows the quantification of various cell morphologies, irregular shapes and even the identification of individual cells that form confluent monolayers by using powerful and flexible segmentation algorithms. Furthermore, Oufti offers post-processing features that enable the identification of differential growth behavior among single cells, e.g. abnormal exponential growth, slow or fast growing cells. The assignment of these observations to cell physiology is, however, often difficult as the molecular causes can be difficult to assess. Next to Outfi, the recently released software MicrobeJ provides a framework for intensity, size and morphology measurements, as well as septa, foci, pole and organelle analyses from microscopy images (Ducret, Quardokus and Brun 2016). The obtained data can be processed and also visualized, with a strong focus on integrated tools for data integrity verification. Besides these generalized software solutions, highly specialized tools have been developed as well. For instance, the software toolbox Molyso has been specifically designed to process time-lapse images obtained from mother machine experiments for growth studies (Sachs *et al.*^2016^).

In general, the above described software tools allow automated high-throughput analyses of single cell traits from images and have become invaluable for processing the massive data amounts from time-lapse experiments. However, automated image analysis algorithms are still error-prone and careful inspection of segmentation results remains inevitable to date.

Besides image-based approaches, growth of single microbes can also be quantified via physical biomass measurements. Cell mass quantification with high resolution is especially useful for uncovering mechanisms involved in, for example, cell death or responses to physicochemical perturbations (Weng *et al.*^2011^; Zangle and Teitell 2014). Cell mass is either quantified as dry mass or as buoyant mass. The dry mass of living single cells can be determined with quantitative phase imaging (Popescu *et al.*^2014^). This technique is based on optical interferometry and enables the differentiation of the refractive index of the cell and the non-aqueous cell content (Popescu *et al.*^2008^). Spatial light interference microscopy is a prominent quantitative phase imaging technology that provides highly sensitive data on a spatial scales from micrometers to millimeters and temporal scaling from seconds to days (Mir *et al.*^2011^). Mir *et al*. applied this technology to profile biomass and to quantify specific growth rates of individual *E. coli* cells that grew on agarose pads. Specific growth rates varied between cells, which demonstrated the individual contributions to the macroscopic biomass increase of a population (Mir *et al.*^2011^). An advantage of quantitative phase imaging is the simultaneous analysis of several cells, regardless of whether the cells are adherent or form biofilms. Suspended microbes are not analyzable with this technology due to artifacts arising from movement. Hence, different technologies are required for quantifying single cell masses in suspension. Godin *et al.* used a suspended microchannel resonator for measuring buoyant masses of individual suspended microbes and human blood cells. The suspended microchannel resonator consisted of a cantilever with an integrated microfluidic channel in an on-chip vacuum (Godin *et al.*^2007^; Weng *et al.*^2011^). Cells were rinsed through the microfluidic channel and detected by the small frequency change of the cantilever's resonance frequency due to the cell. The frequency change is induced by the density difference between the cell and the medium, which directly corresponds to the cell's buoyant mass (Godin *et al.*^2007^; Weng *et al.*^2011^). This particular technology provided subfemtogram-level mass resolution, which makes it applicable to smallest microbial cell types. The technology was applied for studying individual yeast cells in flow-through configuration and for time-related mass analyses of trapped yeast and bacterial cells retained with mechanical barriers (Godin *et al.*^2007^; Bryan *et al.*^2010^; Weng *et al.*^2011^). As an important result, Bryan *et al.* revealed a cell density increase before bud formation of yeast. Such observations can significantly support the understanding of how cells coordinate growth, division and cell cycle progression (Bryan *et al.*^2010^). Suspended microchannel resonators were also applied for measuring the biomass of single marine bacteria (Cermak *et al.*^2017^). The knowledge of biomass composition and contribution by various taxonomic groups provided an estimate of the total marine biomass. With the aid of nutrient flux models, the analyses of single cells might be used to estimate biomass and carbon fluxes in the world's oceans.

The investigation of individual cell growth uncovered the characteristics of distinct physiological growth states of populations. The stationary growth phase of microbial populations is indicated by a constant optical density after nutrient depletion (Monod 1950). This can be for two reasons: cells arrest growth or an equilibrium state between growing and lysing cells is reached (Gefen *et al.*^2014^). Gefen *et al.* monitored single *E. coli* cells that reached starvation conditions in order to distinguish between the two proposed mechanisms. The majority of investigated cells showed arrested growth, while the remaining cells lysed (max. 7%) or grew extremely slowly (max. 5%) (Gefen *et al.*^2014^). The absence of growth was thus verified as the main cause for the stationary growth phase during starvation. Furthermore, the individual metabolic activity of cells during starvation was characterized over several hours by time-resolved investigations of protein synthesis (Gefen *et al.*^2014^). Interestingly, non-growing bacteria maintained a constant metabolic activity over several days under starvation conditions. These investigations proved that the metabolic activity of *E. coli* in the stationary growth phase is not restricted to a small subpopulation of slowly growing cells, but is homogeneously distributed among individuals during extended periods of starvation (Gefen *et al.*^2014^).

#### Biological membrane organization

**Figure 6.**
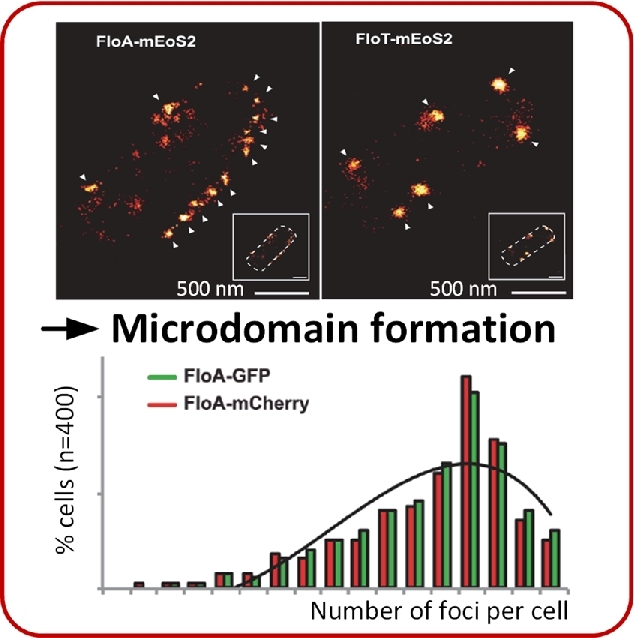
Microdomain formation in growing cells of *Bacillus subtilis*. The two proteins FloA and FloT arrange locally in distinct microdomains in *B. subtilis* cells. Protein localization was revealed by photoactivated localization microscopy of mEoS2-labelled proteins (top). Variability in foci formation of FloA is indicated by fluorescence labeling with green fluorescent protein (GFP) or the red fluorescent protein mCherry (bottom). (Adapted from Schneider *et al*. 2015.)

Microbial membranes are asymmetric and heterogeneous, dependent on their lipid as well as protein composition (Lingwood and Simons 2010; Elani *et al.*^2015^). Biological membranes are among the most important features of cellular life, as they allow the active differentiation of the cell from its surrounding. Microbial growth can only be accomplished when the biological membranes within the cell are intact. In this context, the organization of biological membranes is important as it allows the correct positioning and thus functioning of transport proteins. Membrane-associated proteins are organized in microdomains that are enriched by lipid assemblies called lipid rafts. Those lipid rafts represent a kind of compartmentalization (Schneider *et al.*^2015^). Specific proteins exhibit higher activities when they are arranged in lipid rafts and are thus more efficient (Lopez and Kolter 2010; Schneider *et al.*^2015^). Currently, the existence of lipid rafts in living cells is accepted, but their occurrence in the microbial cytoplasmic membrane was controversially discussed in the past (Munro 2003; Shaw 2006). One reason is the small size of lipid rafts and the difficulty of visualizing them by microscopy technologies. The existence of lipid rafts in eukaryotic microbes was first discovered by *in vivo* single cell studies (Wachtler, Rajagopalan and Balasubramanian 2003). Lipid rafts were visualized by using a fluorescent probe that forms specific complexes with membrane proteins, which was detected with fluorescence microscopy. Later, the existence of microscale domains with equal structures and functions to eukaryotic lipid rafts was proven in bacteria (Lopez and Kolter 2010). Schneider *et al.* investigated the diversity of lipid rafts in individual *B. subtilis* cells (Fig. 6; Schneider *et al.*^2015^). Distinct lipid rafts were responsible for regulatory tasks in cellular membranes. This diversity of functionalized microdomains facilitates the strategic organization of membrane connected signaling networks in a cell. Schneider *et al.* (2015) concluded that bacteria are organized in a more complex way than expected, as bacterial membranes were originally thought to be homogeneous, compartment-free structures. The complexity of membrane-related processes can now be investigated from a completely new perspective.

#### Cell shape adaptation

Cells are able to adapt their shape to spatial restrictions. The adaptability of cell shape to confined cultivation spaces was investigated with a microfluidic device containing microchannels with different heights (Maennik *et al.*^2009^). The device had several adjacent growth chambers, which were connected by microchannels with decreasing diameters. *Escherichia coli* cells were able to actively swim through channels up to widths equal to their own diameter. Cells passed through channels with diameters smaller than their own by growing into the channel entry. The newborn cell in the channel exhibited a smaller diameter than the mother and was able to traverse the small channel and propagate into the next growth chamber (Maennik *et al.*^2009^). *Escherichia coli* cells are obviously able to adapt their size to promote the colonization of their environment. *Escherichia coli* cells that grew in the channels with smaller heights than their own diameter exhibited anomalous broadening shapes compared with the typical rod-shaped morphology (Maennik *et al.*^2012^). Interestingly, these irregularly shaped cells divided into rod-shaped daughter cells of equal size. This demonstrates that cell volume partitioning is robust and accurate during division, even when the cell morphology is temporarily impaired.

A specific definition of cell geometry was achieved by using agarose pads with polydimethylsiloxane ceilings that contained imprinted microchambers. Single *E. coli* cells grew in these chambers with prescribed shapes such as crescents, zigzags, sinusoids and spirals (Takeuchi *et al.*^2005^). The unusually shaped cells were motile and retained their shape after release (Takeuchi *et al.*^2005^). A similar microfluidic device was used to study the adaptation of Min protein dynamics in *E. coli* cells exhibiting anomalous shapes (squares, rectangles, triangles and circles) (Wu *et al.*2015a). Min proteins oscillate from pole to pole over the length of the cells and are responsible for the accurate localization of the cell septum before division in many bacteria (Wu *et al.*2015a). Wu *et al.* visualized Min oscillation using fluorescent fusion proteins in differently shaped cells in order to determine the influence of cell size and geometry. Min protein oscillations aligned to symmetry axes in the cells regardless of the cell shape. The symmetry axes were oriented in such a way that the total length of the axis was between 3 and 6 μm. The Min proteins predominantly rotated in the cell when the symmetry axes were shorter. Disordered oscillations were observed for longer symmetry axes. Overall, the formation of highly artificial cell shapes enabled discovery of the dependency of Min protein oscillation on geometrical parameters and allowed the study of molecular interactions that are dependent on cell morphology.

**Figure 7.**
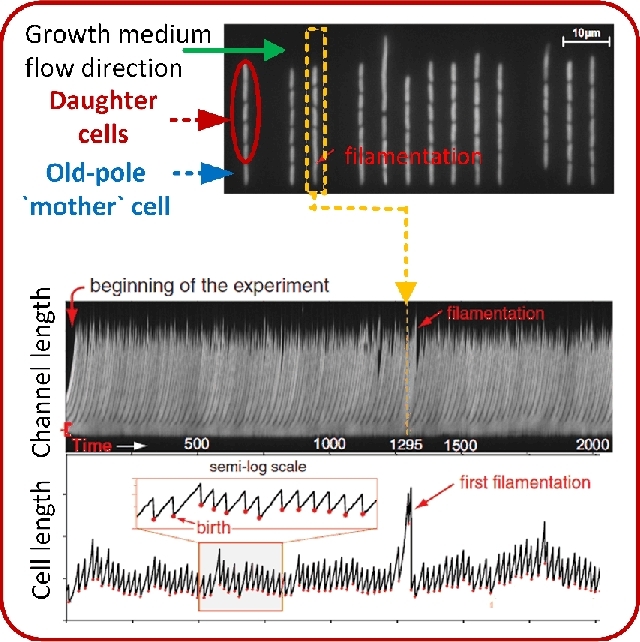
Cell shape maintenance and filamentous growth of *E. coli* is coupled to the replicative age of cells. Filamentation of individual mother cells only occurs at elevated replicative age. (Adapted and republished from Wang *et al.*2010a with permission of Elsevier, Copyright © 2010.)

The filamentous cell shape is a naturally occurring anomalous morphology of bacteria arising without spatial restriction during growth. Bacterial filamentation is a growth phenomenon where cells exclusively grow by elongation without division (Jaimes-Lizcano, Hunn and Papadopoulos 2014). Filamentation is advantageous in waste water treatment, because it is required for flocculation (Aonofriesei and Petrosanu 2007). In biofilms, filamentous bacteria increase the film thickness and roughness. This is disadvantageous in industry because it entails, for example, energy loss in heat exchangers due to fouling (McCoy *et al.*^1981^). Understanding mechanisms provoking filamentous bacterial growth is therefore important. Population-based studies showed bacterial filamentation to be a result of the SOS response (Justice *et al.*^2008^). Individual recombinant *E. coli* cells with a constitutively suppressed SOS response were investigated to validate its role in filamentation (Wang *et al.*2010a). Filamentation rates were reduced in cells with a suppressed SOS response. The suppression of the SOS response also inhibited filamentous cell elongation. Furthermore, experiments with isolated *E. coli* cells that grew in the mother machine revealed a stable, filamentation-free growth for more than 50 generations under steady-state growth conditions. Filamentation was only observed for cells exhibiting an elevated replicative age (Fig. 7; Wang *et al.*2010a). The filamentous phenotype was shown to be reversible in *E. coli* (Probst *et al.*2013b). This was investigated by following growth and morphology over time in a monolayer growth chamber. A filamentous *E. coli* cell of a microcolony was picked with optical tweezers and relocated into the center of a monolayer growth chamber. Astonishingly, the filamentous cells resumed normal growth after a few generations with specific growth rates similar to the original microcolony (Probst *et al.*2013b). The formation mechanism of bacterial filamentation is far from being understood, but the basis has been established. The origin of filamentous bacterial growth will be pursued in future single cell research.

#### Cell wall properties

Cell walls are involved in cell shape maintenance. The cell wall is subject to internal pressure and extracellular restrictions caused by shear forces and spatial boundaries (Takeuchi *et al.*^2005^; Maennik *et al.*^2009^; Pelletier *et al.*^2012^; Amir 2014). The cytosol is crowded due to the large number of macromolecules such as proteins, nucleic acids and carbohydrates (Amir 2014; Nakano, Miyoshi and Sugimoto 2014). Storage of the bacterial chromosome in the restricted space of the cytosol causes entropic forces, which were investigated by Pelletier *et al.* (2012). Individual plasmolyzed *E. coli* cells were loaded into microchannels of the mother machine, digested with lysozyme and lysed by an osmotic shock (Pelletier *et al.*^2012^; Wegner *et al.*^2012^). The rapid cell lysis caused the direct chromosome expansion into the microchannels. Chromosomes were visualized via functional fusion of a fluorescent protein to the nucleoid-associated protein HupA (Marceau *et al.*^2011^). The mechanical properties of the chromosome were quantified by compressing and releasing the chromosome in a closed microchannel using a polystyrene microbead as a piston that was moved by optical tweezers (Pelletier *et al.*^2012^). The forces were approximated with entropic spring models, which depend on compressed chromosome size, the equilibrium length after expansion and characteristic constants. For the first time, the mechanical energy stored in the chromosome was quantified. The mechanical energy amounted to ∼10^5^*k*_B_*T* and repeated chromosome compression required a force of ∼100 pN (Pelletier *et al.*^2012^).

The cell wall has hence to withstand high internal pressures. Simultaneously, the cell wall has to be flexible for adaptation to external forces such as mechanical stresses caused by shear forces (Amir 2014). The resistance of single *E. coli* and *B. subtilis* cells against shear was monitored with cells that were grown in the microchannels of the mother machine. Cells were exposed to filamentation-inducing conditions, which suppressed cell division (Amir 2014). Filamentous cells protruded out of the microchannels into the main trench and were subjected to shear forces, which led to cell bending. Resulting deformations were measured to describe the shape adaptation and recovery capacity of cells. Cells deformed elastically when they were subjected to temporary forces. In contrast, cells deformed plastically when bending forces were constantly applied during growth (Amir 2014). The cell wall composition also affected the bending stiffness of cells (Wang *et al.*2010b). Cell wall mutants of *Escherichia coli* were immobilized on a polyethylenimine-coated coverslip and poly-lysine-coated beads were immobilized on the cell tips. Bending forces were then applied using optical tweezers that manipulated the beads at the cell tips. In this way structural proteins could be identified that contributed to the rigidity of the cell wall (Wang *et al.*2010b).

#### Cell size homeostasis

Cell sizes vary in a narrow range within the same species (Amir 2014; Campos *et al.*^2014^; Jun and Taheri-Araghi 2015). The size of bacteria rarely exceeds the two-fold difference between cell division events when they are cultivated under constant environmental conditions (Jun and Taheri-Araghi 2015). How microbes maintain their size is a central question in microbiology since cellular physiological growth states are still under investigation (Kjeldgaard, Maaloe and Schaechter 1958). Distinct theoretical models were postulated for the underlying intrinsic regulatory mechanisms. It was assumed that cells divide at a critical threshold, dependent on an increase in size, time or volume (Schaechter *et al.*^1962^; Cooper and Helmstetter 1968; Donachie 1968). Validation of these models was impossible with population-based analyses, because they merely deliver averaged data and neglect individual physiological effects (Jun and Taheri-Araghi 2015). Additionally, monitoring a huge number of cell divisions under non-fluctuating environmental conditions was not realizable (Campos *et al.*^2014^).

**Figure 8.**
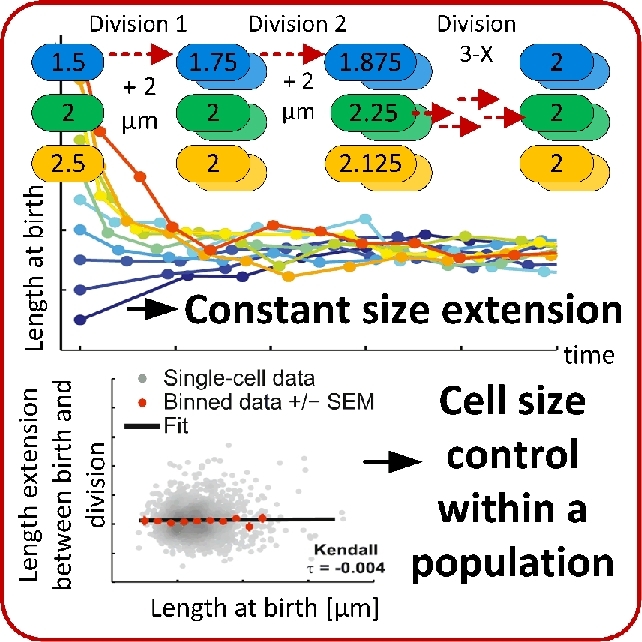
Mechanisms of cell size control. Cell size homeostasis during growth of *E. coli* is achieved by a constant size extension prior to cell division. (Adapted and republished from Campos *et al*. 2014 with permission of Elsevier, Copyright © 2014.)

Time-lapse investigations of cell division in individual *E. coli* and *C. crescentus* cells in a microfluidic device (Ullman *et al.*^2013^) has uncovered the intrinsic principles of bacterial cell size homeostasis (Fig. 8; Campos *et al.*^2014^). Both species exhibited distinct cell division characteristics. *C. crescentus* cells that were shorter than the population average produced daughter cells that were longer than the mother cell. Inversely, cells that were longer than the population average divided into shorter daughter cells. In contrast, *E. coli* cells grew on average to a common length, independent of their original size (Campos *et al.*^2014^). Both species did not divide at a critical size threshold, which refuted the theoretical model of a critical cell size-based bacterial cell size homeostasis. Interestingly, cells of both species constantly elongated before division. This verified the theoretical model of a constant extension mechanism that was hypothesized in the same study (Campos *et al.*^2014^). The constant extension mechanism is based on a constant elongation rate during steady-state growth conditions, in which cells divide when a target length increase is reached (Campos *et al.*^2014^). Experiments with individual *E. coli*, *B. subtilis* and *C. crescentus* cells supported a very similar model (Iyer-Biswas *et al.*^2014^; Jun and Taheri-Araghi 2015). Individual cell divisions of *E. coli* and *B. subtilis* were quantitatively analyzed under seven controlled environmental conditions. Cells added a constant volume before they divided, independent on their original cell size (Jun and Taheri-Araghi 2015). This so-called ‘adder’ behavior was further investigated and linked to the molecular mechanisms of chromosome replication (Wallden *et al.*^2016^). It was found that chromosome replication is activated after the addition of a fixed volume per chromosome. These observations could be translated into a growth and division model for *E. coli* that links cell-to-cell differences in division timing and cell size to variations in specific growth rates. The size of *C. crescentus* cells was found to increase exponentially during cell cycle progression and division occurred when cells reached a multiple of 1.8 of their initial size (Iyer-Biswas *et al.*^2014^). In contrast to mother machine experiments, Iyer-Biswas *et al.* achieved single cell fixation over several generations via inducible cell adhesion. Cells and medium that contained the adhesion inducer were constantly flushed through a microfluidic device. Inducer free medium was supported after cell adhesion, which removed the daughter cells formed that did not adhere (Iyer-Biswas *et al.*^2014^). The authors transferred the experimental findings obtained to a generally valid single cell scaling law for bacteria. Large datasets were implemented into a mathematical model that perceived fluctuations in cell sizes. The established single-cell scaling laws comprise a proportional correlation between the mean division time and the inverse of the mean growth rate, the temperature independency of the mean division-time distribution, and scaling of the coefficient of variation of cell size with the square root of time for a given initial cell size (Iyer-Biswas *et al.*^2014^). With this, a relatively simple, but generally valid mathematical framework was developed for the description of the cell sizing during stochastic growth and division.

To conclude, the cell size threshold model was refuted. A model that is based on a constantly added volume difference was confirmed to describe the mechanisms of cell size homeostasis correctly.

#### Intracellular pH regulation

The intracellular pH (pH_i_) is tightly associated with the cytosolic buffer capacity and linked to the integrity of cell membranes and the membrane potential (Aabo *et al.*^2011^; David *et al.*^2012^; Orij *et al.*^2012^; Bencina 2013). The pH_i_ level affects essential biological processes and enzyme functionalities, regulates metabolic processes such as cellular redox metabolism and molecular transport across membranes, and affects cell vitality and viability (Brett, Donowitz and Rao 2006; Weigert *et al.*^2009^; Aabo *et al.*^2011^; Orij *et al.*^2012^). Earlier population-based attempts to discover the relationship between oscillations in the microbial cell cycle and pH homeostasis were misleading. Karagiannis and Young (2001) revealed that the reported dynamic pH_i_ changes were caused by synchronization steps of the population, which perturbed the cells. Such complex synchronization procedures are obsolete when individual cells are investigated. The pH_i_ of single cells and their cellular compartments are accessible via genetically encoded biosensors such as pHluorin. pHluorin is a green fluorescent protein-derived biosensor that reacts with conformer switching to the surrounding pH (Miesenboeck *et al.*^1998^; Orij *et al.*^2009^; Valkonen *et al.*^2013^). The conformer switch manifests in a changed spatial arrangement of the protein caused by the protonation and deprotonation of its amino acids. The switch induces a detectable shift in fluorescence intensitiy at two distinct excitation wavelengths and can be used as a ratiometric pH probe. The linear response of pHluorin is between pH 5.5 and 8.0, which covers most of the physiological pH_i_ values that have been reported for living microbial cells (Brett, Donowitz and Rao 2006).

pHluorin was used as a probe to measure the pH_i_ of individual *Schizosaccharomyces pombe* cells. Contrary to the results obtained with synchronized populations, the pH_i_ turned out to be constant during the cell cycle of exponentially growing, unperturbed single cells. These results clearly demonstrate how experimental data can be biased by population effects and averaged values.

Maintaining a constant pH_i_ is an indicator for cellular fitness, which in turn affects the quality of biotechnological manufacturing processes (Valli *et al.*^2006^; Valkonen *et al.*^2013^; Valkonen, Penttila and Bencina 2014). Individual *S. cerevisiae* cells were investigated to perform a rational and effective strain improvement strategy regarding lactic acid production (Valli *et al.*^2006^). Accordingly, a correlation between the lactic acid production capability and the pH_i_ maintenance capacity of single cells was established. The pH_i_ was determined by staining with the fluorescent probe carboxy-SNARF-4F AM (SNARF-4F 5-(and-6)-carboxylic acid, acetoxymethyl ester, acetate; Valli *et al.*^2005^, ^2006^). Cells with improved pH_i_ maintenance capacities were more robust against cytosolic acidification (Valli *et al.*^2006^). These cells exhibited higher pH_i_ values and simultaneously higher lactic acid productivities. Cells with improved lactic acid productivities were isolated and applied for further process optimizations (Valli *et al.*^2006^).

#### Heat production

Temperature is an important parameter in industry as it can determine the (economic) success of a bioprocess, since microbial metabolic heat entails a temperature increase in bioreactors that has to be counteracted. Elevated temperatures might cause a deactivation of the cellular biocatalyst, which can be prevented by the installation of cooling technologies (Maskow *et al.*^2010^). Cells emit heat to dissipate excess energy. The magnitude of heat formation depends on the stoichiometry of growth and product formation. Heat emission thus reflects the metabolic activity of the cell (Von Stockar *et al.*^2008^; Maskow *et al.*^2010^). Furthermore, various velocities of biochemical reactions in microbial metabolism are known to be temperature-dependent (Heijnen 1994; Cornish-Bowden 2014). The intracellular temperature thus correlates with biological reactions and functions such as transcription factor binding, mRNA structure, protein folding and membrane permeability (Nobel 1969; John and Weeks 2000; McCabe *et al.*^2011^; Tsuji *et al.*^2013^).

Macroscopic analyses of microbial heat production are commonly based on calorimetric methods combined with theoretical thermodynamic energy balancing (Von Stockar and Briou 1989; Von Stockar *et al.*^1993^; Lechner, Maskow and Wolf 2008). However, the heat power resolution of calorimeters (about 10 mW L^−1^ in a chip calorimeter; Lechner, Maskow and Wolf 2008) is far below the resolution required to detect heat production of single cells (e.g. 7.8 pW for an *E. coli* cell; Lechner, Maskow and Wolf 2008). To our knowledge, only two studies are currently published that report the intracellular temperature quantification in single living microbial cells (McCabe *et al.*^2011^; Tsuji *et al.*^2013^). In the first study, a temperature-sensitive vector was developed. The vector included a promoter that regulated the temperature-dependent *lacZ* expression. Expression of *lacZ*, and thus the production of LacZ (β-galactosidase, EC 3.1.26.12) increased with increasing temperature. LacZ converted fluorogenic substrates into chromophores and its quantity was used to approximate the intracellular temperature (McCabe *et al.*^2011^). The detection range of this method was between 35 and 45°C with an average sensitivity of 0.7°C in *E. coli* cells (McCabe *et al.*^2011^). However, gene expression fluctuations in individual cells were not taken into account with this method.

Next to the genetic thermometer, a cationic fluorescence polymeric thermometer was developed for measuring temperatures inside cells. The fluorescent polymer is spontaneously taken up by *S. cerevisiae* cells and retained within the cytoplasm (Tsuji *et al.*^2013^). The fluorescent polymer exhibited a temperature-dependent fluorescence lifetime. A valid correlation was reported for a temperature range between 15 and 35°C, with a resolution of 0.09–0.78°C (Tsuji *et al.*^2013^).

The development of intracellular thermometers is still at a proof-of-concept stage. Novel insight into biological mechanisms has not yet been obtained by the application of the described techniques. Nevertheless, the presented studies demonstrate fascinating opportunities for the future. One example could be the contribution of cells to global warming, because microbial heat production effects are known to be involved in thawing rates of the arctic permafrost (Schaefer *et al.*^2014^; Hollesen *et al.*^2015^).

### Single cell biochemistry

In this section we review single cell tools and their applications for analyzing biochemical parameters at the subcellular level. Biochemical parameters are involved in functions and mechanisms on all hierarchical cell organization levels, from genome to metabolome (Fig. 2C). Single cell analyses are important for advancing the omics fields, but considering every element of this field would go beyond the scope of this review. Hence, we focus on current technologies and their applications.

#### Genome level

The genome comprises the heritable information of every living organism. Population-based genome studies prevalently provide metagenome data of microbial communities obtained from environmental samples without culturing (Clingenpeel *et al.*^2015^; Oulas *et al.*^2015^). Metagenomes contain data about the entire community without individual differentiation between cells. In contrast, single cell genomics (SCG) makes genome sequences of individual cells accessible, which reveals the metabolic potential of the community (Clingenpeel *et al.*^2015^). SCG therefore links function to phylogeny and assigns gene organization structures to individual genomes within complex environments (Siegl *et al.*^2011^; Stepanauskas 2012; Clingenpeel *et al.*^2015^).

SCG studies have been frequently performed to discover hitherto unknown species of symbiotically living organisms (Siegl *et al.*^2011^; Embree *et al.*^2014^; Kashtan *et al.*^2014^), for establishing genomic blueprints of microbial communities (Probst *et al.*^2015^), and for deciphering genomic heterogeneity in monocultures (De Bourcy *et al.*^2014^). The high resolution of SCG allows comparison of the genetic repertoire of individual population members in monocultures or closely related strains that occupy the same habitat. In contrast, population-based metagenomic analyses often fail to assign analytical information to specific cells due to the inability to cultivate or study them in isolation. In addition, the origin of pooled information is still not precisely traceable with metagenome analyses (Embree *et al.*^2014^). SCG technologies can also be used for analyzing unculturable microbes and especially for deciphering their individual traits (Grindberg *et al.*^2011^; Swan *et al.*^2011^; Martinez-Garcia *et al.*^2012^), which expands the range of applications of population-based genomic methods. As an example, new pathways for microbial CO_2_ fixation were discovered by SCG studies of prokaryotes that occupy subtropical gyres (Swan *et al.*^2011^). Microbial CO_2_ fixation constitutes an alternative way to store the atmospheric carbon surplus (Jiao *et al.*^2014^). Atmospheric CO_2_ affects processes like global warming and climate change, which in turn affect the environmental conditions of natural microbial habitats (Soon *et al.*^1999^). Finding a way to decrease the accumulation rate of CO_2_ in the atmosphere has far-reaching consequences for the ecosphere. Atmospheric carbon storage with maritime chemoautotrophic cyanobacteria is an additional alternative for microbial CO_2_ fixation (Li *et al.*2012b). The fixation mechanisms are not yet completely understood, because methods for the identification of hitherto unculturable microorganisms were lacking for a long time (Li *et al.*2012b). The mechanism of microbial CO_2_ fixation in the dark ocean may also be analyzed with population-based metagenomics. However, single cell technologies enable pure cultures to be provided for further analyses (Swan *et al.*^2011^).

The discovery of new metabolic pathways or related genes of unculturable microbes also supports the exploitation of catalytic potential in biotechnology. The microbes currently used in biotechnological applications represent only a minority of microorganisms present in nature. Only 1% of all bacteria are culturable at present (Lasken and McLean 2014). Hence, the catalytic potential of uncultured microorganisms is still far from being exhausted for industrial biotechnological production processes and will serve as a target for further SCG research in future.

The identification of microbes in microbial communities or hitherto unculturable microorganisms and the amplification of their isolated genomes provide an important basis for SCG (Li *et al.*2012a; Clingenpeel *et al.*^2015^). Technologies for identifying microorganisms in their natural environment include fluorescence *in situ* hybridization (FISH) combined with microautoradiography (MAR) (Kong, Nielsen and Nielsen 2005; Wagner *et al.*^2006^), Raman microspectroscopy (Haider *et al.*^2010^; Li *et al.*2012b; Berry *et al.*^2015^; Zhang *et al.*^2015^) and nanometer-scale secondary ion mass spectrometry (nanoSIMS) (Milucka *et al.*^2012^; Berry *et al.*^2013^; McGlynn *et al.*^2015^). New microbial species and their functional properties in waste water treatment were discovered using FISH-MAR (Kong, Nielsen and Nielsen 2005). In FISH cells are exposed to fluorescently labeled oligonucleotides that specifically bind to rRNA. The cells are then analyzed by fluorescence microscopy and identified according to their staining pattern. MAR technology can be used simultaneously to quantify the uptake of specific molecules. For this purpose, microbes are incubated with radiolabeled substrates prior to analysis and are then exposed to an autoradiographic emulsion. Silver grains are formed on active cells and the amount of silver grains can be correlated with the amount of radiolabeled substrates (Musat *et al.*^2012^). Kong, Nielsen and Nielsen (2005) used FISH-MAR to screen for polyphosphate-accumulating cells in wastewater. New *Actinobacteria* species were thereby identified, which were able to take up and store polyphosphate (Kong, Nielsen and Nielsen 2005). The use of such polyphosphate-accumulating organisms helps to protect wastewater-receiving waters against eutrophication.

An alternative technology for cell identification is nanoSIMS. This is based on mass spectrometry of secondary ions, which are formed by sample bombardment with primary ions. The well-focused beam has a high lateral resolution for sample scanning (Musat *et al.*^2012^). Even subcellular structures like the pigment formed within archaea and proteobacteria from marine sediments can be studied with nanoSIMS (Milucka *et al.*^2012^). However, nanoSIMS is an invasive technology that excludes further analyses of the same cell.

In contrast, Raman microspectroscopy is a non-destructive cell identification method. Molecules inside cells are excited with monochromatic light by a laser. This causes a wavelength shift of the scattered light, which is recorded as a Raman spectrum. The spectrum peaks are characteristic for the composition and conformation of the sample analyzed. Hence, this technology can be used for cell identification or analyses of cell components such as storage compounds (Musat *et al.*^2012^; Majed *et al.*^2012^; Milucka *et al.*^2012^). The non-destructive character of Raman microscopy allows further analyses of the same cell after identification. Berry *et al.* (2015) combined Raman microspectroscopy with FISH to detect heavy water (D_2_O) that was incorporated in distinct active bacterial and archaeal cells. D_2_O-labeled metabolically active cells were isolated with optical tweezers. The genomes of the isolated cells were amplified and subsequently sequenced. The method was applied for identifying individual microbes in the mouse cecum as a proof-of-principle. It is important to understand the compound utilization and composition of the microbial gut community, because it is directly involved in the pathogen defense system. Single cell Raman spectroscopy is also a powerful tool that enables assigning metabolic cell states to genomics (Song *et al.*^2017^). Individual carotenoid-containing cells from Red Sea water samples were isolated with a Raman-activated cell sorting system. The subsequent gene sequencing revealed putative genes responsible for carotenoid biosynthesis. The analysis of metabolism and genome hence allows understanding of the physiological interrelations of biological information and its utilization in cellular catalytic activity.

The amplification of whole genomes or low amounts of nucleic acids contained in a single cell is mostly performed with multiple displacement amplification (MDA). MDA is based on random primer annealing to denatured DNA strands and strand displacement synthesis at a constant temperature catalyzed by a ϕ29 DNA polymerase (Liang, Cai and Sun 2014). MDA provides minor amplification bias, generates long amplicons and has a low error rate of 1 per 10^6^–10^7^ bases (Blainey 2013; Liang, Cai and Sun 2014). Multiple annealing and looping-based amplification cycles (MALBAC) is an alternative single cell gene amplification method (Liang, Cai and Sun 2014). MALBAC is based on multiple cycles of strand displacement pre-amplification with random primers and subsequent amplification by polymerase chain reaction. The amplicons form loops ensuring quasilinear pre-amplification, because the amplification product cannot be used as a new template by the polymerase (Liang, Cai and Sun 2014). Both methods, MDA and MALBAC, were compared for whole genome amplification of single *E. coli* cells with regard to single-nucleotide and copy number variations (De Bourcy *et al.*^2014^). MALBAC was more robust for copy number variant measurements. MDA analysis was more suitable for single-nucleotide variant analyses than MALBAC, since it has a lower amplification-based error rate.

Amplified single cell genomes are mostly sequenced with next generation sequencing technologies, such as the commercially available 454 pyrosequencing (Rothberg and Leamon 2008; Shendure and Ji 2008; Liu *et al.*^2012^) and with Solexa/Illumina (Shendure and Ji 2008) and SOLiD sequencers (Shendure and Ji 2008; Liu *et al.*^2012^). (For a comprehensive description of sequencing technologies and bioinformatics data analysis tools, see Oulas *et al.*^2015^.) In general, next generation sequencing is a reliable, relatively cost-effective and readily available state-of-the-art technology that enables high-throughput sequencing. The capabilities of single cell genome analyses present scientist with new tasks, namely to provide vital single cells suspended in adequate liquid volumes that are free of exogenous DNA. The application of microfluidic lab-on-a-chip devices is one option to separate cells, encapsulate them into droplets, sort them and export them into adequate compartments for subsequent genome analysis (Zhang *et al.*^2017^). However, single cell sequencing technologies require further development in terms of accuracy to unfold their full potential for systems-level analyses of genomes in correlation with physiological data.

#### Transcriptome level

The genome of cells is fixed, meaning that the entire DNA that a cell contains is already determined during cell division. In contrast, the transcriptome is highly dynamic, because the transcript composition varies from cell to cell and from time to time. Transcripts are all kinds of RNA molecules that are synthesized by transcription of DNA, such as tRNA, rRNA, mRNA, as well as the non-coding RNA in the form of introns in eukaryotic cells. Variations in transcript abundance thus affect protein stability, localization, translation and functionality (Pelechano, Wei and Steinmetz 2013).

Transcript variations were detected by investigations of individual yeast cells originating from an isogenic population. The variations were induced by stochastic processes in gene expression and environmental fluctuations (Becskei, Kaufmann and Van Oudenaarden 2005; Colman-Lerner *et al.*^2005^). The biological significance of such cell heterogeneities can be described in more detail by analyzing entire transcriptomes of individual cells (Lidstrom and Meldrum 2003; Wang *et al.*^2015^). The first transcriptome analysis of a single bacterium was performed with *Burkholderia thailandensis* (Kang *et al.*^2011^). Individual cells were isolated with optical tweezers and displaced onto a membrane. Membrane pieces with attached cells were cut and transferred into lysis buffer for further processing. Released mRNA was enriched with a terminator 5΄-phosphate-dependent exonuclease, which selectively degraded rRNA and tRNA. Enriched mRNA was transcribed into circularized cDNA, which was amplified using MDA (Kang *et al.*^2011^). This novel strategy for transcript amplification was chosen because earlier linear and exponential amplification methods required immense laboratory work, had a low reproducibility and had extensive gene expression bias (Kang *et al.*^2011^). The long processing times often resulted in RNA loss due to extremely low transcript contents contained in a single cell and the short half-life of RNA (Gao, Zhang and Meldrum 2011). The circularized cDNA was analyzed by DNA microarray technology; 94–96% of transcripts from single *B. thailandensis* cells were reliably sequenced with this approach and the individual induction and repression of gene product syntheses by the inhibitor glyphosate were detected (Kang *et al.*^2011^).

*Burkholderia thailandensis* cells contain extremely high transcript levels of about 2 pg, which is not representative for the majority of bacterial cells (Kang *et al.*^2011^). Lower amounts of RNA, about 5 fg, are sufficient for sequencing with the RNA sequencing method BaSiC (*Ba*cterial *Si*ngle *C*ell) (Wang *et al.*^2015^). This method is simple to use and requires no specialized equipment, because all sequencing steps can be performed with commercially available kits with minor modifications. Individual cells are separated with a micromanipulator, RNA is isolated with a Zymo RNA Isolation Kit and RNA is translated to cDNA with the One-Direct RNA Amplification System. Resulting cDNA is amplified with the NuGen WT-Ovation One-Direct RNA Amplification System and sequenced by using Illumina's Solexa sequencer. The applicability of the method was validated by transcriptome analyses of individual *Synechocystis* sp. PCC 6803 cells; 82–98% of single cell transcriptomes were sequenced within a processing time of 8 h per cell. The duration of the sequencing process for a single cell transcriptome has to be significantly reduced, because high throughput is required to distinguish between stochastic and significant differences in gene expression. Alternatively, the process could be optimized by parallelization to achieve high throughput and to yield statistically relevant data (Wang *et al.*^2015^).

#### Proteome level

Proteins, in their role as enzymes, are responsible for catalyzing metabolic reactions, replicating DNA, the cellular response to stimuli, the regulation of intracellular biochemical processes and the transport of molecules (Schmid *et al.*^2010^). The entire protein content of a cell at a specific time point represents the proteome. The proteome decisively affects the physiological state and the ability to respond to changing environmental conditions. Interestingly, a correlation between the protein and transcript copy number of any gene was not detected within single *E. coli* cells, although transcriptome and proteome are dependent on the same gene expression mechanisms (Taniguchi *et al.*^2010^). This contradiction was ascribed to different lifetimes of mRNA and proteins. Transcripts in the form of mRNA are mostly degraded within minutes, whereas most proteins have longer lifetimes than the cell cycle. The transcriptome thus reflects the recent transcriptional activity, while the proteome represents accumulated gene expression over the lifetime of the cells (Taniguchi *et al.*^2010^). A single microbial cell contains more than 1000 different protein species depending on environmental conditions (Schmid *et al.*^2010^). A lack of methods for the simultaneous recording of the entire protein content of a single cell makes whole proteome analyses extremely challenging and currently impossible. Nevertheless, parts of the proteome of single cells have already been successfully analyzed.

Enzymes of individual *E. coli* cells were quantified with an enzyme-linked immunosorbent assay and monitored via fluorescence microscopy (Stratz *et al.*^2014^). Individual cells were mechanically trapped between two pillar barriers of a sealable fluidic microchamber. The trapped cells were lysed and enzymes, in this case a β-galactosidase, were bound to antibodies that were previously immobilized by avidin linkers and poly(L-lysine)-g-poly(ethylene glycol) biotin (Stratz *et al.*^2014^). The enzyme quantity was detected by a fluorescence signal, which was emitted after the addition of fluorescein di-β-D-galactopyranoside. The β-galactosidase hydrolyzed fluorescein di-β-D-galactopyranoside into fluorescent fluorescein and galactose. The limit of detection was as low as 200 enzymes. The β-galactosidase concentration depended on the composition of the growth medium and the β-galactosidase abundance in individual *E. coli* cells was variable (Stratz *et al.*^2014^). This demonstrates the phenotypic heterogeneity in the proteome of isogenic populations.

Intracellular proteins can also be localized and quantified with single molecule localization microscopy (SMLM). Stochastic optical reconstruction microscopy and photoactivated localization microscopy are the most important SMLM techniques for protein analyses in single cells. Both technologies are based on the sequential activation of photoswitchable fluorophores (Lukyanov *et al.*^2005^; Bates, Jones and Zhuang 2013). The high spatial resolution of such technologies allows single molecule detection and hence cellular functions can be assigned to specific proteins.

SMLM technologies were used, for example, to describe how genetic material is organized in bacteria (Dame, Wyman and Goosen 2000; Jensen and Shapiro 2003; Strunnikov 2006; Sullivan, Marquis and Rudner 2009; Schwartz and Shapiro 2011; Wang *et al.*2011a; Scolari, Sclavi and Cosentino Lagomarsino 2015; Song and Loparo 2015). Similar to DNA condensation in chromosomes of eukaryotes (Misteli 2007), a global chromosome organization mechanism enables plugging of long DNA in small bacterial cells. The plugging mechanism is related to protein–DNA interactions (Nolivos and Sherratt 2014; Song and Loparo 2015). The contribution of nucleoid-associated proteins (NAPs) and structural maintenance of chromosomes proteins (SMCs) to the bacterial chromosome organization was identified with population-based analyses (Song and Loparo 2015). However, these analyses were not sufficient to decrypt the underlying mechanisms, due to the limited resolution for studying protein localization.

**Figure 9.**
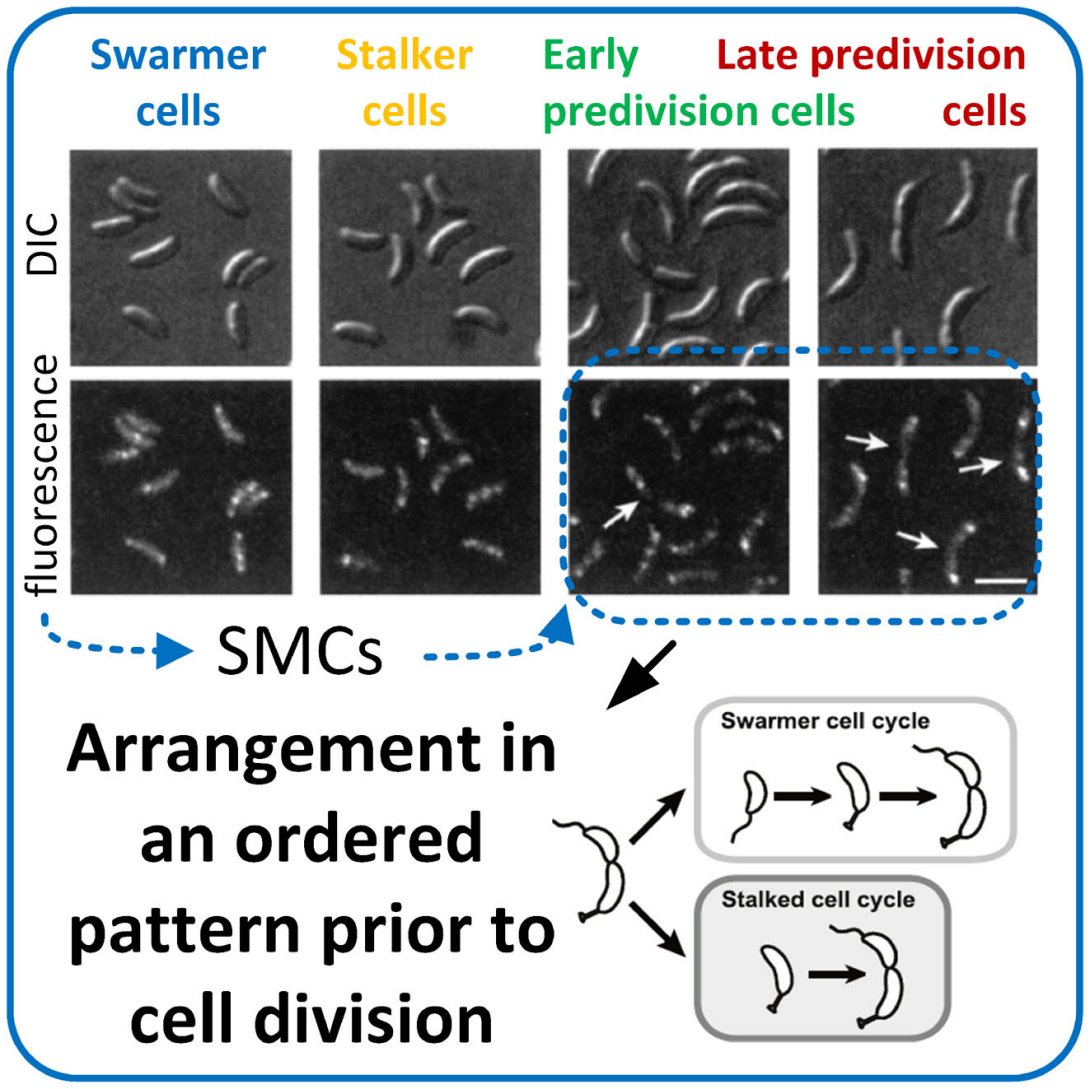
Bacterial chromosome organization in *C. crescentus*. The example shows the structural maintenance of chromosomes proteins (SMCs) that are involved in bacterial DNA condensation. The SMCs arrange in an ordered manner prior to cell division. (Adapted and republished with permission of American Society for Microbiology — Journals, from Jensen and Shapiro (2003); Adapted and republished from Campos *et al*. 2014 with permission of Elsevier, Copyright © 2014.)

Experiments with single *C. crescentus* cells proved that SMCs interact with DNA in an ordered pattern. SMCs aggregated as condensing complex foci in the bacterial chromosome (Jensen and Shapiro 2003). SMC dynamics during the cell cycle and cell division have been successfully discovered in different bacteria (Mascarenhas *et al.*^2002^; Jensen and Shapiro 2003; Strunnikov 2006). SMC foci localized at the cell poles prior to cell division of individual *C. crescentus* cells and immediately disappeared after division (Fig. 9; Jensen and Shapiro 2003). Hence, the polar SMC aggregates dissociated during cell division. In contrast, NAPs were found to form DNA clusters by bridging and wrapping DNA strands. Variable DNA-condensation degrees, which are regulated by NAPs, suggested a smart polymer function for the bacterial nucleoid. This was revealed by analyzing single cells with SMLM (Scolari, Sclavi and Cosentino Lagomarsino 2015). Smart polymers react to external stimuli with large changes in their conformational transition. External stimuli can be small changes in the environmental temperature, pH values or salt concentrations. NAPs thus cause either the extension or the condensation of the chromosome in response to small changes in environmental conditions (Scolari, Sclavi and Cosentino Lagomarsino 2015). Wang *et al.* reported that the NAP H-NS forms compact clusters in the bacterial nucleoid that are separated from each other, which was analyzed with fluorescent fusion proteins (Wang *et al.*2011a). In a further study, the fusion of distinct fluorescence marker proteins to the same NAP was identified to originate from methodological artifacts. The large, discrete clusters were caused by dimerization of fusion proteins (Wang *et al.*^2014^; Song and Loparo 2015). In truth, the abundance of H-NS is much more dispersed than expected. This demonstrates the importance of a deliberated choice of analysis method and the design of control experiments.

**Figure 10.**
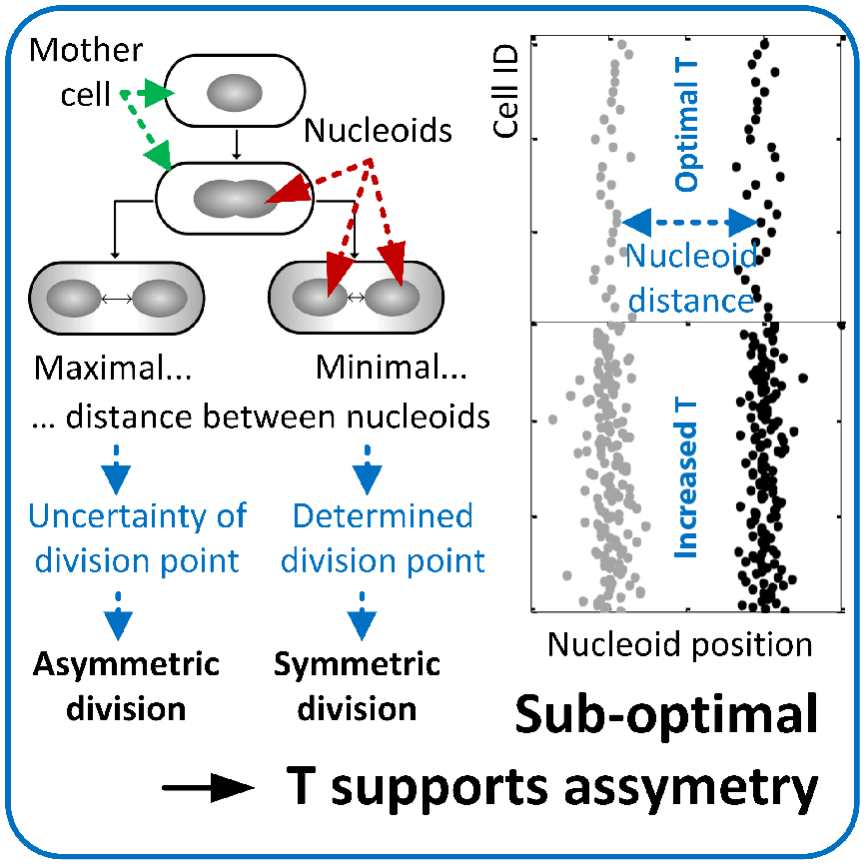
Consequence of nucleoid distances for the symmetry of cell division. Increased mean distances between the nucleoids support asymmetric cell division in *E. coli*. Non-optimal temperature favors increased nucleoid mean distances as well as symmetry breaking during cell division. (Adapted from Gupta *et al*. 2014 © IOP Publishing. Reproduced with permision. All rights reserved.)

The knowledge obtained about the bacterial chromosome organization formed the basis for pursuing single cell studies in other research areas, e.g. studies of cell division, DNA replication and DNA repair (Britton *et al.*^2007^; Simmons *et al.*^2008^; Gupta *et al.*^2014^; Uphoff and Kapanidis 2014; Stracy *et al.*^2015^). *Escherichia coli* cells divide in a morphologically symmetric manner under optimal growth conditions, while the intracellular material is stochastically distributed between the dividing cells (Yu and Margolin 1999; Gupta *et al.*^2014^). The division of individual *E. coli* cells was followed for nine generations by automated time-lapse microscopy of monolayer colonies in order to investigate cell aging (Stewart *et al.*^2005^). Cells with inherited old poles (the end of the cell pre-existing from a previous division) grew more slowly, produced fewer daughter cells and had an increased probability of dying. Interestingly, morphological characteristics were identical between cells with old and new poles (Stewart *et al.*^2005^). This means that the seemingly identical cells resulting from the same division exhibit functional asymmetries and proved that *E. coli* is prone to aging. *Escherichia coli* is not, however, capable of compensating those functional asymmetries under external stress exposure (Gupta *et al.*^2014^). Gupta *et al.* visualized morphologically asymmetric cell divisions and an increased uneven inheritance of matter by analyzing fluorescently tagged NAPs (Fig. 10). A temperature increase stimulated the smart polymer function of the nucleoid, which resulted in nucleoid expansion in dividing cells. In consequence, the mean distances between the nucleoids increased. The uncertainty of the point of cell division consequently increased, because more space was available to set the division point. The division points varied from cell to cell, caused morphological asymmetry and hence resulted in phenotypic heterogeneity within isogenic populations (Gupta *et al.*^2014^).

The decryption of unequal cell division mechanisms might enable a specific manipulation of phenotypic intrapopulation heterogeneities in future. The ability to control phenotypic heterogeneity has significant consequences for biotechnological processes, because it can influence the robustness to process conditions (Delvigne and Goffin 2014), as well as the survival of microbial populations in fluctuating natural environments (Bishop *et al.*^2007^; Chakraborty and Li 2011).

#### Metabolome level

The metabolome encompasses substrates, intermediates and products of the entire metabolism with molecular masses of typically less than 2 kDa (Zenobi 2013). This includes amino acids, organic acids, fatty acids, carbohydrates, nucleotides, adenosine triphosphate (ATP) as energy carrier and redox equivalents like NAD(P)(H) and FAD(H) as cofactors (Brett, Donowitz and Rao 2006). The intracellular metabolite composition changes at time scales between milliseconds and seconds (Zenobi 2013). Monitoring of the metabolome condition is challenging because of its dynamic character. The various chemical molecule classes within the metabolome even complicate accessing the entirety of metabolites. Metabolite quantification of a single cell is especially challenging, because the minute sample volume corresponds to the dimensions of the cell (size 1–10 μm, volume 1 fL to 1 pL) and very low total molecule concentrations prevail inside a cell (from a few hundred up to 10^10^ molecules per cell) (Schmid *et al.*^2010^; Zenobi 2013).

Fluorescently labeled metabolites, biosensors or mass spectrometry (MS)-based methods have been developed for metabolite quantification at the single cell level. The quantification of substrate uptake can be simply realized in real-time by tagging substrates with fluorescent labels. Glucose and toluene were, for example, tagged with *N*-(7-nitrobenz-2-oxa-1,3-diazol-4-yl)amino (NBD) resulting in the fluorescent molecules 2-NBD-2-D-glucose and 3-NBD-3-toluene. Those molecules were used for analyzing individual glucose and toluene uptake of *E. coli* cells (Natarajan and Srienc 1999; Straeuber *et al.*^2010^; Nikolic, Barner and Ackermann 2013). The attachment of fluorescent labels to metabolites might, however, affect their biochemical function (Zenobi 2013). Biosensors, which provide correlations between metabolite concentrations and readouts such as fluorescence intensity levels, do not affect the metabolite function. Biosensors are even suitable for real-time detection of intracellular metabolite concentrations (Van Engelenburg and Palmer 2008). Various types of biosensors have been developed for quantifying different metabolites. Such sensors can be based on the production of fluorescent proteins that is under the control of a promoter, which is induced by the presence of specific metabolites. Nicotinamide adenine dinucleotide (NADH), hydrogen peroxide or several amino acids were, for example, quantified with these kinds of sensors (Belousov *et al.*^2006^; Binder *et al.*^2012^; Mustafi *et al.*^2012^; Knudsen, Carlquist and Gorwa-Grauslund 2014; Mustafi *et al.*^2014^). Other biosensors, such as circularly permuted fluorescent protein-based and Förster resonance energy transfer-based sensors, exploit conformational changes or interactions of fluorescent proteins caused by binding of specific metabolites (Berg, Hung and Yellen 2009; Yaginuma *et al.*^2014^; Boersma, Zuhorn and Poolman 2015). The conformational changes cause switches in the fluorescence spectrum or intensity. Although the application of biosensors is limited when the simultaneous reporting of different metabolites in one single cell is intended, the analyses of metabolites with biosensors are in general highly sensitive and enable a quantitative, time-resolved monitoring of metabolite levels in living single cells (Zenobi 2013).

**Figure 11.**
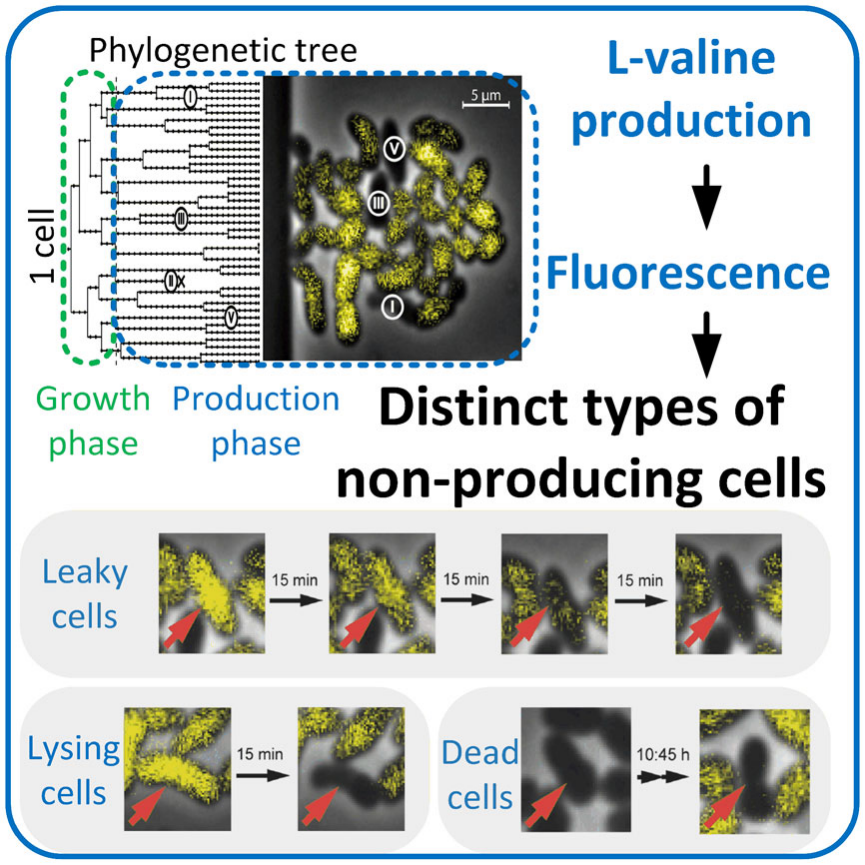
Metabolic activity of single cells. The example shows the connection between metabolic activities and amino acid production capacities in *C. glutamicum* cells. The occurrence of distinct non-producing phenotypes in a heterogeneous culture was revealed by time-resolved productivity studies employing a genetically encoded fluorescent metabolite sensor. (Adapted from Mustafi *et al*. 2014.)

This was realized in a biotechnological context, where biosensors were applied for process development. Valuable metabolites in biotechnological processes include, for example, amino acids, which are mainly produced by *C. glutamicum* (Hermann 2003; Eggeling and Bott 2015). Interestingly, the production of the amino acid L-valine by *C. glutamicum* cells varies with distinct cell viability states (Fig. 11; Mustafi *et al.*^2014^)*.*L-Valine production, which was initiated by a medium change, was decoupled from growth in the L-valine producer strain. The interplay between growth, physiology and metabolite production was investigated via single cell cultivations in monolayer growth chambers (Mustafi *et al.*^2014^). Growth and viability were detected by time-resolved live cell imaging and intracellular L-valine production was monitored with a fluorescent biosensor. L-Valine production was heterogeneous during the production phase and the heterogeneity distribution width increased during cultivation (Mustafi *et al.*^2014^). Some cells were viable and productive in the beginning of the production phase and then suddenly lysed. Other cells did not start to produce, because they were either dead or dormant. Another type of non-producing cell continued to grow without L-valine production, although the preferred growth substrate was not delivered after medium transition (Mustafi *et al.*^2014^). The occurrence of these distinct cell types illustrates a complex regulation during the absence of growth substrates, which underlies the formation of non-producing cells. The non-producing cells might negatively influence the productivity of industrial biotechnological production processes. The reduction of such phenotypes might prospectively lead to the enhancement of industrial process efficiency.

**Figure 12.**
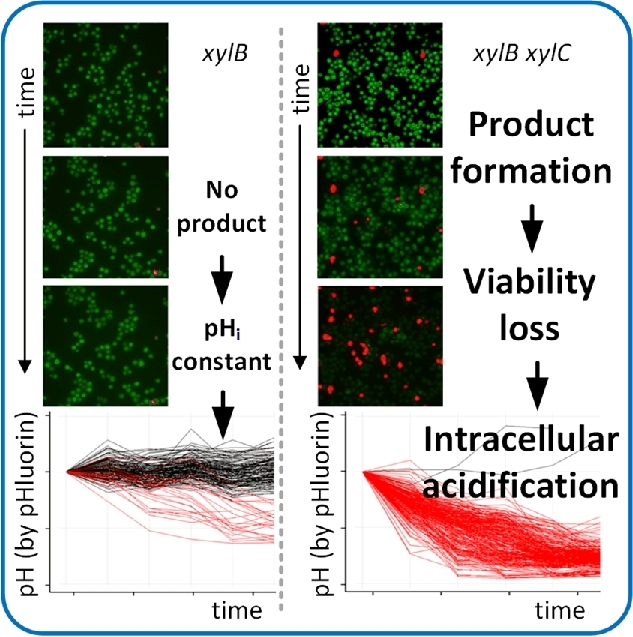
Monitoring intracellular properties of single microbes. The example shows D-xylonate-producing *S. cerevisae* cells that were equipped with pHluorin (green) for monitoring the intracellular pH. The cells were additionally stained with propidium iodide (red) for visualizing non-viable cells. It was found that cells lose their viability as a result of intracellular acidification caused by D-xylonate accumulation. (Adapted and republished from Nygard *et al*. 2014 with permission of Elsevier, Copyright © 2014.)

Beyond the exploitation of the natural metabolite production of microbes, new synthetic metabolic pathways can be created that do not exist in nature. This adds novel functions to well-known gene networks or improves the activity of the target pathways (Fritz *et al.*^2010^). Single cell microbiology allows a precise characterization of novel pathway properties. The non-native D-xylonate production pathway from D-xylose in *S. cerevisiae* is an example of a synthetic gene network (Toivari *et al.*^2012^; Nygard *et al.*^2014^). D-Xylonate is an organic acid that serves as an important platform chemical for the production of biomass-derived plastics or as a concrete additive (Chun *et al.*^2006^; Toivari *et al.*^2010^). Cells exhibiting the synthetic pathway accumulate D-xylonate intracellularly (Toivari *et al.*^2012^). Nygard *et al.* hypothesized that this product accumulation causes a vitality/viability loss during D-xylonate production, which is highly undesirable for an industrial production process. Mechanistic characteristics of the new metabolic pathways were analyzed in order to test this hypothesis (Fig. 12). The lactone ring opening reaction prior to D-xylonate production was identified as the rate-limiting step, revealed with time-resolved analyses of individual cells containing a biosensor (Nygard *et al.*^2014^). These restrictions of the heterologous production pathways can now be used for specific process optimization by debottlenecking metabolic conversions via targeted genetic engineering.

MS-based technologies constitute a good alternative for the simultaneous analysis of several metabolites, because they provide quantitative information and deliver structural descriptions of molecules at the same time (Lottspeich and Engels 2006). The most commonly used ionization technologies for analyzing small amounts of metabolites are matrix assisted laser desorption/ionization (MALDI) (Urban *et al.*^2010^; Passarelli and Ewing 2013) and electrospray ionization (Mizuno *et al.*^2008^; Heinemann and Zenobi 2011). MALDI-MS technologies enable the simultaneous sensitive, label-free detection of numerous metabolites. Nevertheless, the application of MS for single cell analysis is currently restricted to one measurement time point as the analysis compulsorily consumes the cell. Also the full ionization of minute amounts of target molecules, which are usually present in chemically complex solutions, is challenging. Low-abundant molecules could be concentrated with repeated ion accumulation in ion trap mass spectrometers to overcome this challenge (Si *et al.*^2017^). This method was successfully validated for concentrating ATP up to 22-fold. High-density microarrays for MS (MAMS) were developed to aliquot small volumes of solutions or suspensions. This technique enabled high-throughput metabolite analyses of single cells with a detection limit of 100 amol to 10 fmol (Urban *et al.*^2010^; Ibanez *et al.*^2013^). A total of 26 intracellular metabolites of individual *S. cerevisiae* cells were simultaneously measured with this approach (Ibanez *et al.*^2013^; Zenobi 2013). Ibanez *et al.* further validated the biological information that can be obtained with MAMS. Intrinsic metabolic cell-to-cell differences that emerge from cell size, cell age or cell cycle stage were described by the metabolite analysis of individual cells. Two different phenotypes were thus identified in an isogenic *S. cerevisiae* population by MS-based measurements of the intracellular levels of the glycolytic metabolite fructose-1,6-bisphosphate. In addition, differences in metabolic network regulations were disclosed in single *S. cerevisiae* cells upon perturbation with 2-deoxy-D-glucose, which blocks glycolysis. The shift of metabolic fluxes was determined by correlating metabolites pairwise. Perturbed cells exhibited a more active pentose phosphate pathway. Metabolites that were too far away from the metabolic entry point of 2-deoxy-D-glucose were not affected by the perturbation (Ibanez *et al.*^2013^). Such metabolite correlations of individual cells enabled description of metabolic regulatory mechanisms at a high resolution and provide a glimpse of future applications of single cell metabolomics.

To conclude, the combination of single cell analyses with standard population-based analyses enables a mechanistic understanding of cellular biology (Table 1). Several fundamental biological questions have already been addressed with this approach and some of the complex underlying biological mechanisms are currently under investigation. The answer to one biological question mostly gives rise to several new ones, often in relation to other physiological parameters. Thus, a network of knowledge about the idealized cell in its environment is established step by step and non-idealities are minimized or describable for the first time. This is in accordance with the demand of synthetic biology that claims abstractable and quantifiable biological elements that can be used to synthesize novel functional cell systems.

**Table 1.** Examples for new biological concepts obtained with single cell microbiology which would not have been possible from population-based microbiology.

Technology for cultivation/analysis	Analysis/data	New concepts	References
High-throughput microscopy	Growth rate and gene expression	Intrapopulation heterogeneity is beneficial for surviving e.g. heat stress	Levy, Ziv and Siegal (2012)
Microfluidic growth chamber, agarose pads, microscopy	Growth and gene expression	Lag phases during diauxic shifts result from persistent cells that continue to grow slowly resulting in a delay in biomass monitoring	Boulineau *et al.* (2013), Solopova *et al.* (2014)
Microfluidic cultivation device, microscopy	Intracellular pH and gene expression	A cross-protection between antibiotics and other stressors enhances cell survival	Mitosch, Rieckh and Bollenbach (2017)
Microscopy	Viability, bioluminescence and population cell density	Variabilities in quorum sensing mechanisms are related to individual bioluminescence and biofilm formation	Anetzberger, Pirch and Jung (2009)
Microscopy	Bioluminescence, cell size and gene expression	Autoinducer concentration correlates with population cell densities. Autoinducers are involved in the formation of intrapopulation heterogeneity	Anetzberger, Schell and Jung (2012)
Agarose pad, microfluidic monolayer growth chamber, Envirostat, time-resolved microscopy	Specific growth rates	Individual cells that experience optimal growth conditions have higher specific growth rates compared with population scale analyses	Dusny *et al.* (2015)
Microscopic cell monitoring in a microfluidic device	Elongation rate	During stationary growth phase conditions, the majority of cells do not grow; a minority of cells lyse or grows slowly	Gefen *et al.* (2014)
Agarose pad, microfluidic monolayer growth chamber, Envirostat, time-resolved microscopy	Division rates, division angles and division symmetry	Non-optimal growth conditions are better compensated by specific growth rates than by morphological characteristics	Dusny *et al.* (2015)
Microscopy, photo-activated localization microscopy	Gene expression	Bacteria are organized in a more complex way than expected as membrane proteins are arranged in microdomains as a kind of compartmentalization	Schneider *et al.* (2015)
Mothermachine, microscopy	Cell shape	Filamentous growth is dependent on the replicative cell age	Wang *et al.* (2010a)
Monolayer growth chamber, optical tweezers, microscopy	Growth and morphology	The filamentous growth phenotype is not stable but reversible	Probst *et al.* (2013b)
Agarose pads, time-lapse microscopy	Cell length, elongation rate and relative growth rates	Bacterial cells elongate in a constant manner before cell division	Campos *et al.* (2014)
Mother machine, time-lapse microscopy	Growth rate, elongation rate, cell size, generation time	Bacterial cells add a constant volume before they divide	Taheri-Araghi *et al.* (2015b)
Microfluidic cultivation device, microscopy	Growth rate, cell volume, generation time	Chromosome replication is initiated after the addition of a fixed volume per chromosome	Wallden *et al.* (2016)
Microfluidic flow chamber, fluorescence-activated cell sorting, time-lapse microscopy, biosensor	Cell cycle, intracellular pH level, cell length	Exponentially growing cells maintain a constant intracellular pH level during the cell cycle	Karagiannis and Young (2001)
Flow cytometry, microscopy, pH staining	Metabolite secretion, intracellular pH level	Cells with improved intracellular pH maintenance capacities exhibit higher intracellular pH levels during lactic acid production resulting in production rates	Valli *et al.* (2006)
Microscopy	Intracellular temperature	Proof of concept of temperature measurement technologies for measuring heat production in single cells	McCabe *et al.* (2011), Tsuji *et al.* (2013)
Microfluidic device, microscopy	Growth, growth arrest and gene expression	The metabolic activity in the stationary growth phase is not restricted to a small subpopulation of slowly growing cells but is homogeneously distributed among individuals	Gefen *et al.* (2014)
Monolayer growth chamber, microscopy, biosensor	Intracellular metabolite concentration, gene expression, viability, growth	Non-producing cells exhibit distinct variability states	Mustafi *et al.* (2014)
Envirostat, microscopy	Gene expression	A promoter system was proven to be extremely sensitive to carbon-catabolite repression, which was previously severely underestimated with population-based analyses	Dusny and Schmid (2016)
Single cell genomics	Genome information	Discovery of hitherto unknown species of symbiotic living organisms and pathways of non-cultivable organisms	Siegl *et al.* (2011), Grindberg *et al*. (2011), Swan *et al.* (2011), Martinez-Garcia *et al.* (2012), Embree *et al.* (2014), Kashtan *et al.* (2014)
Single molecule localization microscopy	Molecule localization	Bacterial chromosome organization mechanisms exist, similar to DNA condensation in chromosomes of eukaryotes	Strunnikov (2006), Wang *et al.* (2011a), Song & Loparo (2015)
Immunofluorescence microscopy	Molecule/protein localization	Proteins interact with DNA in an ordered pattern and arrange at the cell poles prior to cell division	Jensen and Shapiro (2003)
Microfluidic flow chamber, time-lapse microscopy, nucleoid staining	Molecule localization, morphological (a)symmetry, cell segmentation	Symmetry breaking during cell division depends on the mean distance between bacterial nucleoids. Division symmetry is temperature dependent	Gupta *et al.* (2014)

## TECHNICAL BIAS IN SINGLE CELL MICROBIOLOGY

The examples discussed above demonstrate the importance of single cell microbiology and its contribution to obtaining new biological insights beyond populations. However, single cell technologies also have technology-specific restraints. There is little knowledge about how technical bias affects microbial physiology. Very few experimental studies shed light on these aspects despite obvious physiological effects imposed by the technologies of single cell cultivation and analysis. From our experience, bias from single cell technologies is commonly discussed amongst researches, but often omitted in scientific publications. We therefore think it is important to discuss some major aspects of this topic within the frame of this article.

### Microhabitats

The most commonly occurring complications in single cell microbiology are growth arrest, loss of viability, changing cell morphologies or altered gene expression patterns upon the introduction of cells into the artificial cultivation microhabitat. The reasons for such phenomena can be manifold and their identification often requires tedious trial-and-error experiments. Attention should be given to the choice of the device material in the first place to identify the origin of these complications.

Biocompatible polymers and adhesives, primarily polydimethylsiloxane, are typically used for manufacturing microfluidic devices. Secreted molecules or medium compounds can be sequestered into the polymer, as polymeric substances such as polydimethylsiloxane adsorb small hydrophobic molecules and proteins (Halldorsson *et al.*^2015^). Polymers might be prone to monomer, oligomer or additive leaching as well. The interaction of such leached molecules with cells potentially entails adverse effects on cellular physiology (Regehr *et al.*^2009^). The magnitude of leaching is much more pronounced in microdevices compared with macroscopic cultivations, because microstructured devices have high surface-to-liquid volume ratios. Material compatibility tests should be performed during the device design stage or whenever leaching is suspected to affect microbes. Molecule leaching or sequestration in the device material can be avoided by altering surface characteristics. Inherently hydrophobic polymers such as polydimethylsiloxane are often hydrophilized by plasma treatment during manufacture of the microdevice (Bodas and Khan-Malek 2007; Zhou *et al.*^2012^). Priming of the microfluidic network with sacrificial molecules, e.g. with albumin-spiked growth medium or Tween, can effectively passivate surfaces and restore surface biocompatibility after activation (Turner *et al.*^2010^; Taheri-Araghi *et al.*2015a). Alternatively, biocompatible aqueous materials such as agarose gels can be considered for microdevice manufacture (Moffitt, Lee and Cluzel 2012). Direct cell-surface contact further enhances the exposure of cells to leaching substances and might promote adverse cellular behavior (Petrova and Sauer 2012). Surface contact triggers regulatory cascades associated with cell adhesion, which usually remain inactive in liquid cultures (Geng *et al.*^2014^). Non-contactless trapping of single microbes with, for example, negative dielectrophoresis serves here as an alternative (Fritzsch *et al.*^2012^).

Microdroplets used for cultivation occupy an exceptional position among the single cell microhabitats. The aqueous cultivation volume is typically confined by a second immiscible continuous phase instead of solid channel walls (Joensson and Andersson Svahn 2012). Technical bias in microdroplets primarily arises from adsorption effects at the liquid–liquid interface (Roach, Song and Ismagilov 2005). Proteins and hydrophobic molecules accumulate at the interface and are depleted in the aqueous cultivation volume owing to the hydrophobic nature of the continuous phase. This effect can be significant due to the high surface-to-volume ratio of the microdroplets. In addition, the physiology of the confined cells is influenced by traces of the continuous phase that can dissolve in the aqueous droplet. Surfactants for droplet stabilization also play a critical role, as they were shown to induce detrimental processes such as cell lysis in eukaryotes (Clausell-Tormos *et al.*^2008^).

### Cell trapping and manipulation technologies

Single cell retention, relocation or isolation technologies potentially impose technical bias on microbial physiology. Directed cell manipulation technologies such as optical manipulation, acoustic trapping and electrokinetic trapping involve the exposure of single microbes to a focused light beam, ultrasound or an electric field, respectively (Eriksson *et al.*^2007^, ^2010^; Evander *et al.*^2007^; Kortmann *et al.*2009a). The impact on the cellular physiology depends on the magnitude of field intensity, exposure time and the properties of the biological system. Exposure times and field intensities should be as low as possible to avoid cell damage. Optical tweezers, for example, are only suitable for quickly relocating living cells over short distances within microhabitats, but not for longer trapping of single microbes. Extended optical manipulations of *E. coli* resulted in a rapid viability loss (Probst *et al.*2013b). Growth immediately ceases after the shortest exposure times to the light beam for some microorganisms that are particularly sensitive to light irradiation, such as *C. glutamicum* (C. Probst, personal communication). Single cell manipulation technologies based on electric fields, such as dielectrophoresis, entail inherent technical bias as well. Local resistive heating, called Joule heating, in dielectrophoresis trapping structures arises from the passaging current through the growth medium. In consequence, temperatures inside the trapping structures can be several degrees above the actual chip temperature, which entails physiological responses of trapped cells (Jaeger, Mueller and Schnelle 2007). Quantitative knowledge on the magnitude of heating is necessary for the effective compensation of joule heating with temperature control systems (Rosenthal *et al.*^2015^). Joule heating compensation has already allowed long-term single cell cultivations of several yeast and bacteria species in negative dielectrophoresis traps without technology-borne effects on microbial physiology (Dusny *et al.*^2015^).

It is of course always advisable to perform control experiments in order to identify and circumvent biological artifacts that might arise from technical bias during cell trapping and manipulation. Reliable comparative experiments can involve population-based controls, but also single cell experiments with complementary cultivation technologies. A simple and cheap variant for control experiments can, for instance, be performed with agarose pads as single cell growth supports (Young *et al.*^2012^).

### Mode of cultivation

Unexpected cellular behavior can be imposed by the mode of cultivation. Many microfluidic devices enable the continuous perfusion of single cells with growth medium, which allows controlling and changing of the chemical composition in the single cell microenvironment (Di Carlo, Wu and Lee 2006; Kortmann *et al.*2009a; Gruenberger *et al.*^2012^). Perfusion, however, creates a highly artificial situation for the trapped cells, where secreted substances are immediately removed due to the continuous medium flow. In contrast, secreted molecules accumulate to various extents during population-based cultivations. Low quantities of molecules accumulate in the microenvironmental cell surroundings even in continuous chemostat fermentations on a population scale. In consequence, microbial strains show impaired growth behavior when they rely on the extracellular presence of specific secreted substances. Such an impaired growth behavior was described for *B. subtilis* cells, which were cultivated with minimal medium in a microfluidic perfusion device (Taheri-Araghi *et al.*2015b). Growth was restored upon introduction of sterile-filtered, cell-free minimal medium obtained from the late exponential or stationary phase of previous cultivations. Growth did not arrest when complex medium was applied in the single cell perfusion system. The authors attributed this observation to secreted ion carrier molecules that are essential for an effective uptake of extracellular metal salts and hence for cell growth (Langdahl and Ingvorsen 1997). Similar observations were made by our group when phototrophic microorganisms were cultivated in single cell perfusion systems (unpublished). Growth was immediately interrupted upon perfusion of cells with fresh minimal medium, but could be restored by the addition of minor amounts of cell-free medium from a former batch culture. These observations in microfluidic systems demonstrate how microbes shape their environment. The application of conditioned minimal medium or the addition of essential nutrients can provide information about the necessity of secreted molecules for growth (Taheri-Araghi *et al.*2015a).

### Analytics

Technical bias from the analysis technique itself might also result in biological artifacts, which leads to wrong conclusions on the cellular mechanism of interest. The validation of single cell experiments with carefully designed control experiments is especially necessary when novel analysis technologies are used. Single cell experiments are presumably more strongly affected by technical bias than experiments with whole populations, because disturbances from analysis technologies do not equally affect all cells in populations. Biological artifacts can hence be compensated by measuring averages from a large number of individuals.

Optical methods are presumably the most prominent technologies for analyzing physiological dynamics in single microbial cells. Fundamental biological mechanisms have been unraveled in single microorganisms with time-lapse brightfield or phase contrast microscopy complemented with fluorescence imaging of fluorescent dyes or proteins (Lee *et al.*^2012^; Young *et al.*^2013^; Vasdekis and Stephanopoulos 2015). Although optical analysis technologies are defined as being non-invasive, this is not always the case. Light-induced phototoxicity can affect all types of microorganisms. Phototoxicity comprises a number of potential mechanisms, in which the formation of highly reactive oxygen species or radicals is probably the most damaging (Zipfel, Williams and Webb 2003; Davies 2004). Even white illumination used for standard brightfield/phase contrast imaging can entail photo-induced effects on microbial metabolism (Woodward, Cirillo and Edmunds 1978; Merbt *et al.*^2012^). Photo-induced physiological effects can be especially critical when high energy UV light from filament-based white light lamps is applied. Appropriate filters for filament-based lamps or LED illumination with a defined light spectrum prevents such UV-induced effects.

Things become more critical when fluorescence imaging is used. The physiology of many microbial cell types is susceptible to changes or damage by fluorescence excitation light (Tinevez *et al.*^2012^). A non-linear negative correlation between the dose of green fluorescent protein excitation light and single cell division times was demonstrated for *E. coli* (Jun and Taheri-Araghi 2015). The minimization of phototoxic effects can be accomplished by reducing light exposure time or excitation light intensity or by increasing detector sensitivity via, for example, detector pixel binning. Excellent guidelines on avoiding pitfalls during fluorescence imaging of live cells are already available (Dixit and Cyr 2003; Frigault *et al.*^2009^). The basic principles for avoiding technical bias of fluorescence imaging can also be applied for microbes, although most work is based on cell cultures. Another aspect of fluorescence imaging is the application of fluorescent proteins or dyes. Genetically encoded fluorescent proteins can impose a metabolic burden for microbes (Wendland and Bumann 2002). Fusion of fluorescent proteins to cellular proteins allows monitoring of the abundance, localization and dynamics of specific proteins, but might influence the native physiological function of the tagged protein (Shaner, Steinbach and Tsien 2005). The delay in signal occurrence due to synthesis and maturation times of fluorescent proteins in biological system has to be considered as well when dynamic cellular processes are analyzed (Hebisch *et al.*^2013^). Chemical dyes can also be applied, for example to selectively stain cell organelles or cellular components or to determine cell viability. However, chemical dyes often intercalate DNA or impair the function of the stained molecules (Terai and Nagano 2013). These considerations suggest that controls are mandatory for identifying and eliminating analysis-borne technical bias on microbial physiology.

We are convinced that extensive control experiments help to identify and circumvent biological artifacts that might arise from the technical bias described, regardless of whether bias is caused by the microhabitat, cell manipulation, mode of cultivation, or the analysis technology used.

## FUTURE CHALLENGES OF MICROBIAL SINGLE CELL ANALYSIS

Single cell analysis has changed the way we look at microbial physiology. However, the capability of current single cell technologies is limited for the investigation of some specific research questions. An extensive discussion of these limits would go beyond the scope of this review. As such, we focus on a few representative examples.

Culturability of diverse microorganisms present in the natural and technical ecosystems is in general an important issue in single cell microbiology. The majority of published single cell studies focus on the analysis of domesticated model bacteria and yeast. The cultivation and analysis technologies are often specifically tailored for their analysis. Cultivation and analysis of their natural relatives raise new issues in terms of technological compatibility.

Very few single cell studies with obligate anaerobic microorganisms have been published yet, although these microbes are highly abundant in nature and important for medical and technical processes (Steinhaus *et al.*^2007^; Fievet *et al.*^2015^). The main challenge for cultivating obligate anaerobic microorganisms is posed by the necessity for absolute oxygen absence from the microhabitat. The complete desorption of oxygen from growth medium and from polymers used for microfabrication is difficult to realize. However, anaerobic conditions could be established by placing the complete analysis system and its periphery in an anaerobic enclosure.

The maximal possible cultivation time of microbes is another important aspect. Long-term cultivations are necessary to follow dynamic processes in cells that have a very slow metabolism. This can be problematic when technical restraints impede long-term single cell cultivations, such as evaporation or irreversible and adverse changes of the microhabitat material. Nevertheless, technical solutions for rarely investigated microbes such as obligate anaerobes or slow-growing microbes will emerge with growing interest in single cell microbiology.

Technological development constitutes the main driver for progress in single cell analysis. Novel and sophisticated analytical technologies provide ever deeper insights into cellular mechanisms of microbes. In addition usable technology is the key, not the existence of proof-of-concept technology alone (Andersson and van den Berg 2006). Many essential cellular parameters have already been successfully determined and others will hopefully follow in the upcoming decade (Love *et al.*^2012^; Hammar *et al.*^2015^; Krone *et al.*^2016^). This knowledge opens the door to new approaches for improving the efficiency of microbes in a technical context.

## CONCLUSIONS

Single cell microbiology has added a new conceptual dimension to microbiology. In this article, we discussed the advances and exciting possibilities of single cell technologies and demonstrate how they enable formulating and testing new hypotheses. Single cell technologies enable the analysis of cellular mechanisms in individual microbes with spatiotemporal resolution unbiased by population effects. Causal links between environmental parameters and cellular physiology can be established because of the precisely controlled microenvironment within microfluidics. Microbiologists, biotechnologists and biochemical engineers working with microbes can benefit from applying these technologies for answering research questions hidden in the bulk of microbial populations. Numerous studies demonstrate how single cell technologies can be used for approaching biological questions concerning mechanisms in microbial metabolism, its regulatory circuits and interconnections, gene expression, protein synthesis, homeostasis in size, shape and intracellular properties, cell-to-cell communication, as well as mass and energy transfer. We can learn from these analyses how natural microbial concepts are set up in order to efficiently perform biological functions, like growth, product formation, survival or adaptation to environments in natural and artificial ecosystems. This information can be used to identify, understand and abstract the constraints that control microbial functional modules in order to maximize the efficiency of their biological functioning. These considerations clearly show that technological developments are the main driver for obtaining novel biological insights with single cell microbiology. However, some studies demonstrate the importance of considering technical bias from novel single cell cultivation and analysis technologies. This reminds us to critically question the results obtained with carefully designed control experiments. Single cell analysis has matured to be an integral part of microbiology and future technological advances will give us new and exciting insights into the properties and mechanisms of the smallest functional unit of life, the microbial single cell.
